# Retinoblastoma and beyond: pediatric orbital mass lesions

**DOI:** 10.1007/s00234-024-03517-6

**Published:** 2024-12-27

**Authors:** Zoran Rumboldt, Doris Dodig, Paolo Galluzzi, Ivan Brumini, Rebekah Clarke, Sumit Singh, Andrea Rossi

**Affiliations:** 1 Department of Diagnostic and Interventional Radiology, Faculty of Medicine, University of Rijeka, Braće Branchetta 20, Rijeka, 51000 Croatia; 2 Department of Radiology and Radiological Science, Medical University of South Carolina, Charleston, SC USA; 3 Telemedicine Clinic, Barcelona, Spain; 4 Department of Neuroimaging and Neurointervention, Siena University Hospital, Siena, Italy; 5 Division of Pediatric Radiology, University of Texas Southwestern Medical Center Children’s Health, Dallas, TX USA; 6 Department of Neuroradiology, Istituto Giannina Gaslini, Genoa, Italy

**Keywords:** Retinoblastoma, Pseudoretinoblastoma, Venolymphatic malformations, Rhabdomyosarcoma, Infantile hemangioma, Neuroblastoma, Langerhans cell histiocytosis, Dermoid

## Abstract

Various space occupying lesions can arise in the orbit, ranging from developmental anomalies to malignancies, and many of the diseases occurring in children are different from the pathologies in the adult population. As the clinical presentation is frequently nonspecific, radiologic evaluation is essential for lesion detection and characterization as well as patient management. While orbital masses may in some cases involve multiple compartments, a simple compartmental approach is the key for the diagnosis on imaging studies, and MRI is the modality of choice. This pictorial review presents the most common and characteristic non-emergent pediatric orbital lesions, stressing their MRI and CT appearances, including specific differentiating features. The lesions are subdivided into 4 compartments: intraocular, intraconal, extraconal, and orbital walls. Retinoblastoma, Coats disease and persistent fetal vasculature; optic pathway glioma and lymphovascular malformations; rhabdomyosarcoma, infantile hemangioma, neurofibroma and lymphoma; neuroblastoma, leukemia/myeloid sarcoma, Langerhans cell histiocytosis and dermoid are reviewed in their respective compartments.

## Introduction

The orbit is a relatively small but complex anatomical space with bony walls containing the globe, extraocular muscles, neurovascular structures, fat and the lacrimal apparatus, from which a wide spectrum of possible pathologic processes can arise. While some diseases overlap with those encountered in adults, many orbital masses are unique to pediatric patients, such as retinoblastoma and neuroblastoma [1–3]. Clinical symptoms and signs, which include diplopia, visual impairment and eye pain, leukocoria, strabismus, restricted ocular motility and periorbital swelling, may point to the likely pathology but are often insufficient to reliably differentiate between orbital lesions, particularly in young children [1, 2]. Biopsy may be performed, but is frequently avoided, especially for suspected retinoblastoma, because of a risk of seeding [4–6]. Radiology therefore plays a vital role in the diagnosis and management of orbital lesions.

Due to its superior soft tissue contrast, accurate depiction of the orbital compartments and lack of ionizing radiation, MRI is the preferred imaging method [1, 2]. Dedicated protocols include thin-section (3 mm or less) T1 and T2-weighted sequences (axial and coronal), diffusion imaging with ADC maps, and postcontrast images; STIR sequence is commonly acquired and fat saturation may be utilized for extraocular diseases; high resolution 3D sequences (T2 and T1-weighted) are particularly helpful for evaluation of intraocular lesions and perfusion MR imaging (primarily and preferably arterial spin labelling technique, ASL) is useful in certain cases. Although not a part of the specific MRI protocols, 3D FLAIR of the brain with fat suppression is very sensitive for orbital abnormalities, most conspicuously the intraocular and those involving the optic nerve. A dedicated anesthesia team may be necessary for the acquisition of diagnostic quality MR images, especially in younger pediatric patients [2, 3]. US with high-frequency probes (usually performed by ophthalmologists) is typically the initial imaging modality for intraocular pathology and may also be used for characterization of vascular lesions, while CT is nowadays almost exclusively performed when assessment of the bony walls is needed [1, 3]. Traumatic and nontraumatic orbital emergencies, for which CT is very often the initial imaging modality, are beyond the scope of this article. FDG-PET (and possibly other nuclear medicine studies) may be beneficial in certain clinical settings.

While this review is by no means all-inclusive and complete, it describes the characteristic imaging findings and specific differentiating features of the common pediatric orbital masses using the systematic approach based on the intraorbital compartments [1, 7], and we hope that it will be a helpful aid in daily clinical practice.

## Intraocular compartment

1. Retinoblastoma (RB)
2. Coats Disease
3. Persistent Fetal Vasculature (PFV)

*Retinoblastoma* (RB) is the most common intraocular tumor in children. Mean age at clinical presentation is 2 years for unilateral forms and 1 year in bilateral forms, 95% are diagnosed under the age of 5 [4, 8–10]. RB can be sporadic (60%) or hereditary with bilateral disease nearly always due to an inherited abnormal *RB1* tumor-suppressor gene, while unilateral neoplasm is sporadic in 85% of cases. Germline *RB1* mutation also predisposes to intracranial midline neoplasms of the pineal or/and suprasellar region (trilateral RB) [4, 8–11].

Leukocoria (white pupillary reflex) is the most common presenting feature and can be seen by the naked eye, strabismus and decreased vision are also frequent clinical findings. RB is one of the few malignancies that is routinely treated without prior histopathologic or genetic analysis [4–6]. Biopsy is avoided because of a risk of seeding, while genotyping using liquid biopsy from the aqueous humor is currently being developed [6]. Retinoblastoma is curable. If detected while still confined to the globe and if there are no metastatic risk factors, the child will nearly always survive following appropriate treatment. Current eye-sparing interventions include selective intra-arterial chemotherapy and intravitreal chemotherapy, photocoagulation, thermoablation, cryotherapy, and temporary implant of radioactive iodine for radiation therapy. Systemic chemotherapy is generally reserved for bilateral and advanced disease [4, 9]. Enucleation is usually performed for eyes showing clinical or imaging high-risk features.

Imaging includes color fundus photography, ultrasonography, fluorescein angiography and optical coherence tomography (OCT, which provides high resolution cross-sectional image of the retina and permits detection of clinically invisible lesions). MRI confirms the diagnosis, determines the orbital extent of the disease, and defines extraorbital involvement; evaluates response to therapy and potential post-treatment complications when the eye becomes clinically inaccessible [9, 10]. European Retinoblastoma Imaging Collaboration has published imaging guidelines for diagnostic evaluation of retinoblastoma: MRI T2-weighted and T1-weighted sequences without and with contrast agent, without fat saturation; high spatial resolution with section thickness ≤ 2 mm and in-plane pixel size ≤ 0.5 × 0.5 mm. For optimal detection of optic nerve invasion, the image plane (axial and sagittal oblique) should align with the distal (1 cm) end of the nerve, just posterior to the lamina cribrosa. The brain should always be imaged in retinoblastoma patients for analysis of midline structures to depict trilateral retinoblastoma or leptomeningeal spread [12].

**Fig. 1:**
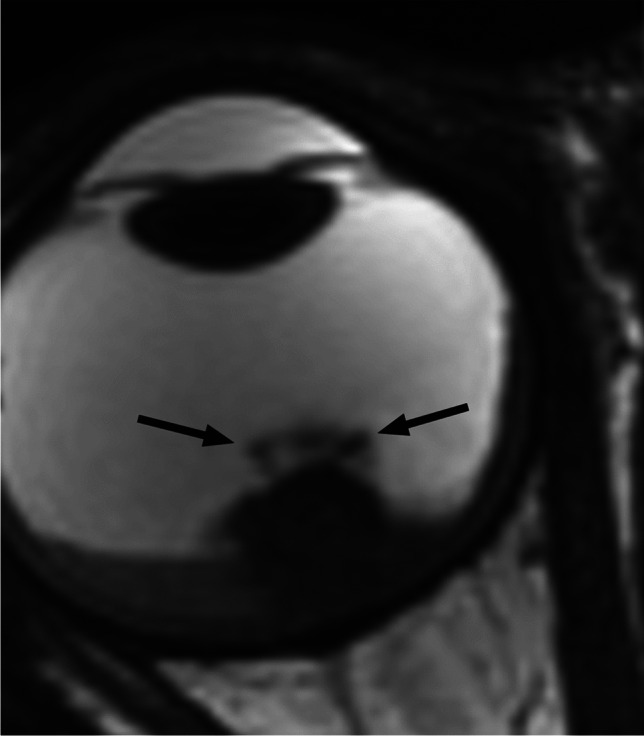
High resolution axial T2-weighted image of the globe shows retinoblastoma with endophytic growth into the vitreous chamber, including vitreous seeding (arrows)

**Fig. 2:**
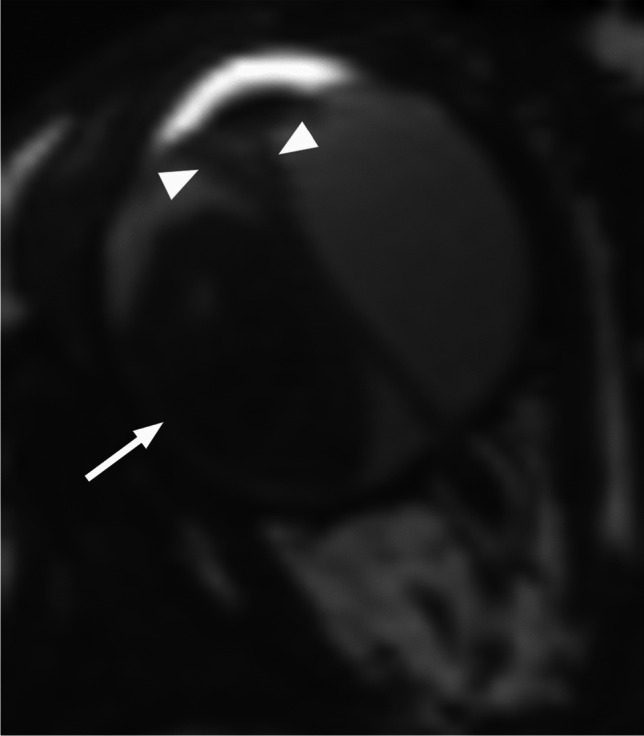
Exophytic growth of retinoblastoma (arrow) with a prominent V-shaped retinal detachment (arrowheads) on axial T2-weighted image through the globe

**Fig. 3:**
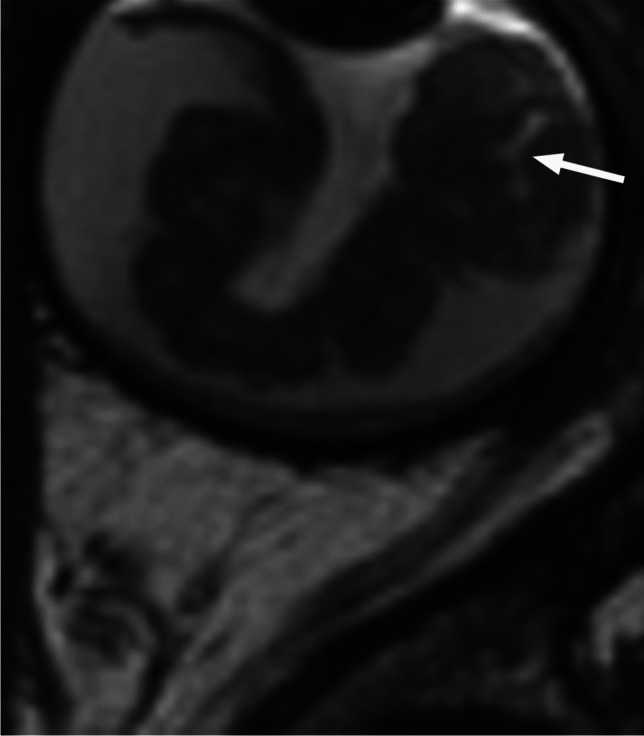
High resolution axial T2-weighted image of the globe reveals retinoblastoma with diffuse, infiltrative growth along the detached retina. There is tumor-retina folding with enclosure of the vitreous (arrow)

RB originates in the retina and can display endophytic growth into the vitreous chamber (Fig. 1); exophytic growth into the subretinal space (Fig. 2); or diffuse, infiltrative growth along the retina (Fig. 3). Most lesions demonstrate both endophytic and exophytic growth and can cause retinal detachment as well as vitreous and subretinal tumor seeding [4, 8, 9].

**Fig. 4:**
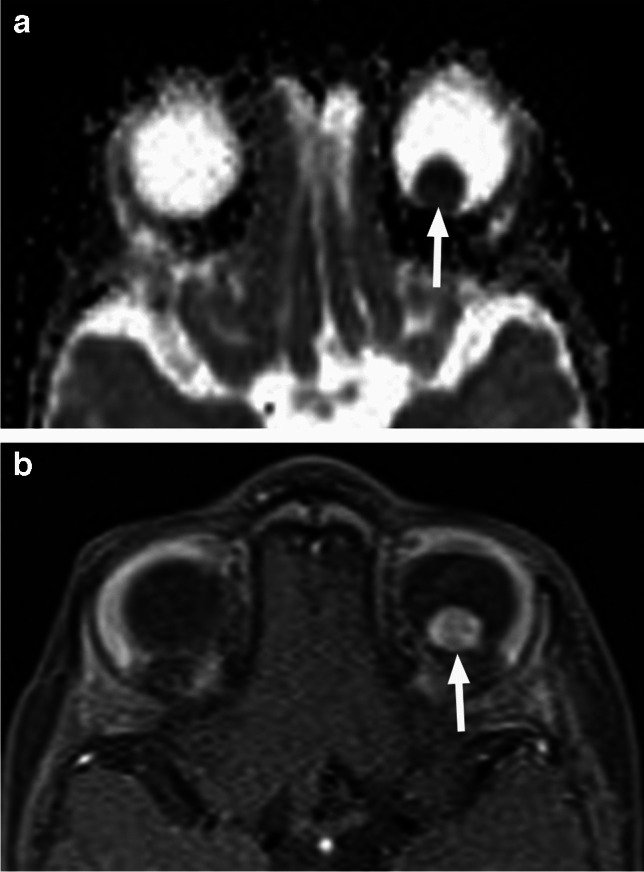
Left eye retinoblastoma in an infant. Nodular intraocular lesion (arrows) shows low diffusivity (low signal intensity, hypointense to the brain) on axial ADC map (**a**) and enhancement on corresponding postcontrast T1-weighted image with fat saturation (**b**)

**Fig. 5:**
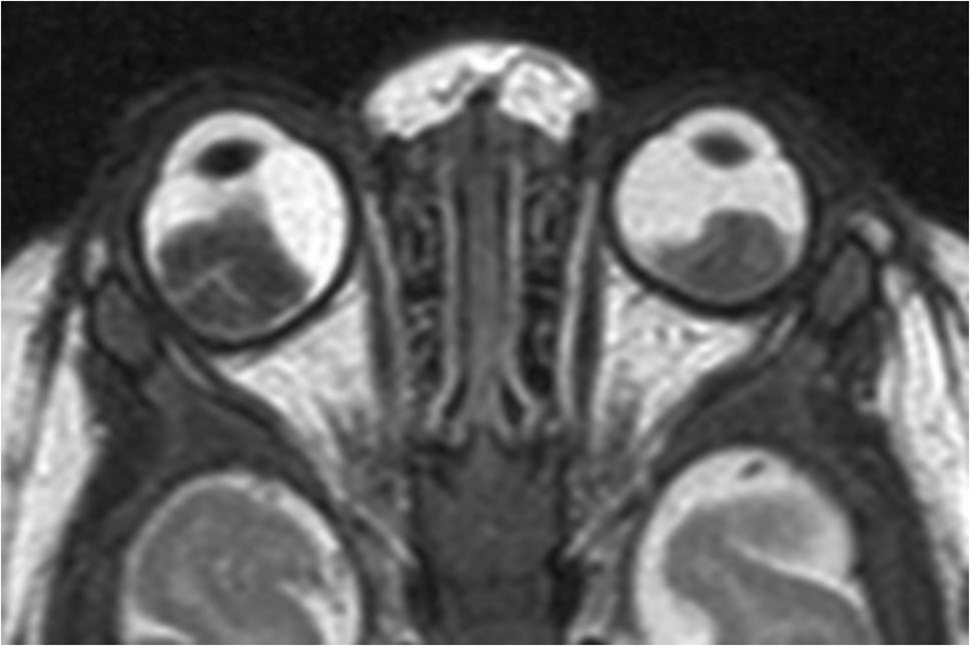
Bilateral retinoblastoma. Axial T2-weighted image of the orbits demonstrates intraocular hypointense masses arising from the retina of both eyes

**Fig. 6:**
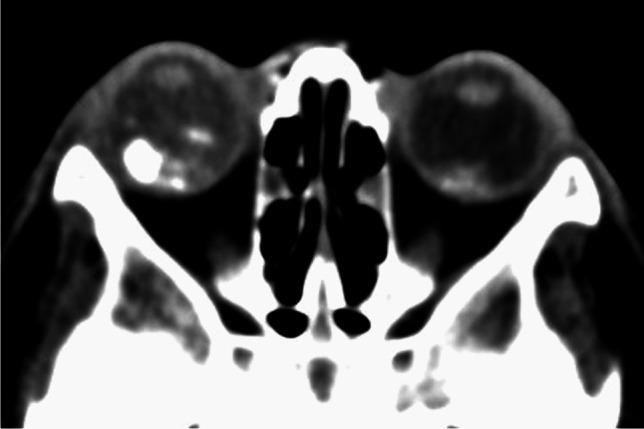
Bilateral retinoblastoma. There are right greater than left intraocular masses containing calcifications on this axial CT image of the orbits with soft tissue algorithm and window

On MRI RB typically appears as an intraocular mass with irregular contours, T2 hypointense (Figs. 1, 2 and 3) and of intermediate T1 signal (slightly hyperintense compared to the vitreous) with markedly low signal on ADC maps (low diffusivity) and heterogeneous contrast enhancement (with non-enhancing necrotic parts of the mass) (Figs. 4 and 5). The affected globe is frequently enlarged. Foci of signal loss, best seen on SW MR sequences, correspond to calcification. Calcification is a key diagnostic feature (present in around 95% of cases) (Fig. 6), which is reliably detected by ocular ultrasound. CT is no longer indicated because of ionizing radiation (especially in patients with *RB1* mutation who have an increased risk of second primary malignancy) and no added diagnostic value [4, 9, 10, 12, 13]. Retinal detachment, when present, is typically V-shaped [10] (Figs. 1 and 3).

**Fig. 7:**
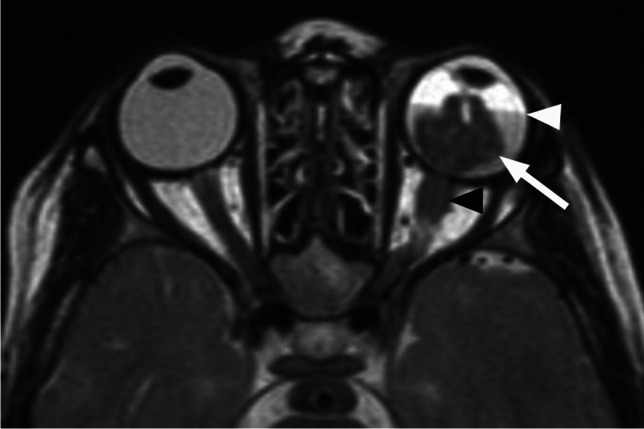
Retinoblastoma with postlaminar optic nerve invasion (PLONI). Axial T2-weighted image shows left intraocular hypointense mass (arrow) with retinal detachment and subretinal fluid level (white arrowhead). There is also thickening of the retrobulbar optic nerve (black arrowhead)

**Fig. 8:**
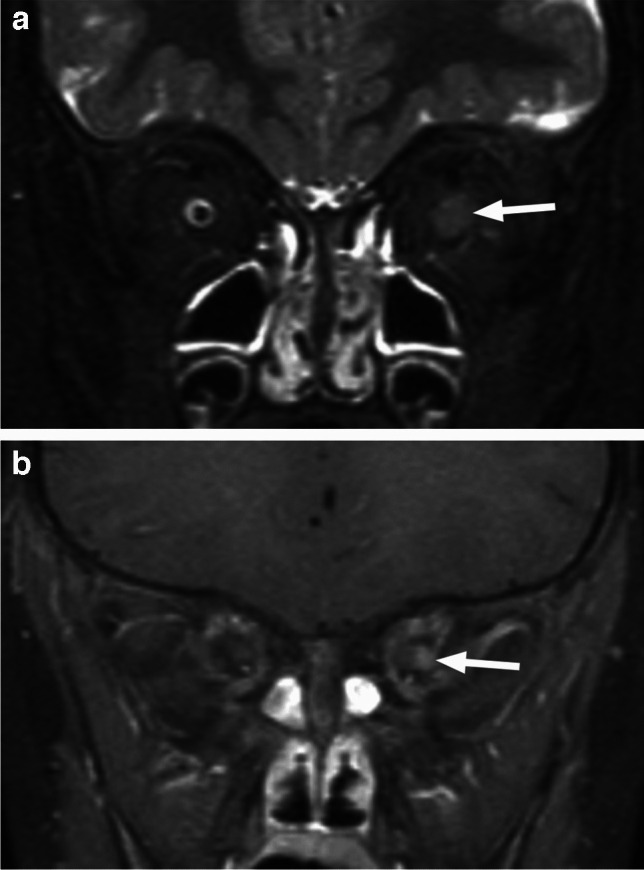
Coronal STIR image (**a**) shows thickening and increased signal intensity of the retrobulbar left optic nerve (arrow). There is also enhancement of the intraorbital nerve (arrow) on a more posterior coronal postcontrast T1-weighted image with fat saturation (**b**). The findings are consistent with PLONI in this patient with retinoblastoma of the left eye

**Fig. 9:**
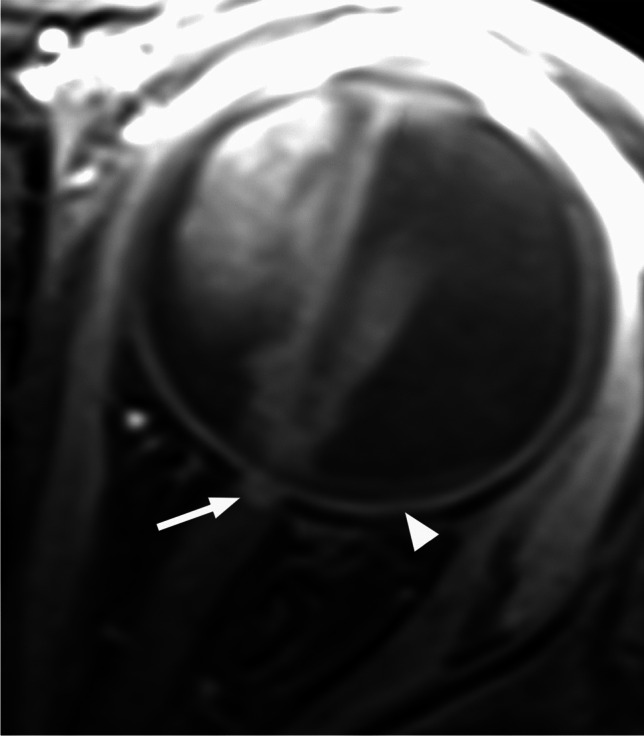
Axial postcontrast T1-weighted image of the globe in a patient with retinoblastoma demonstrates postlaminar optic nerve enhancement (white arrow) that is similar with the adjacent uninterrupted choroid (black arrow), indictive of inflammation. There was no nerve infiltration at histology

**Fig. 10:**
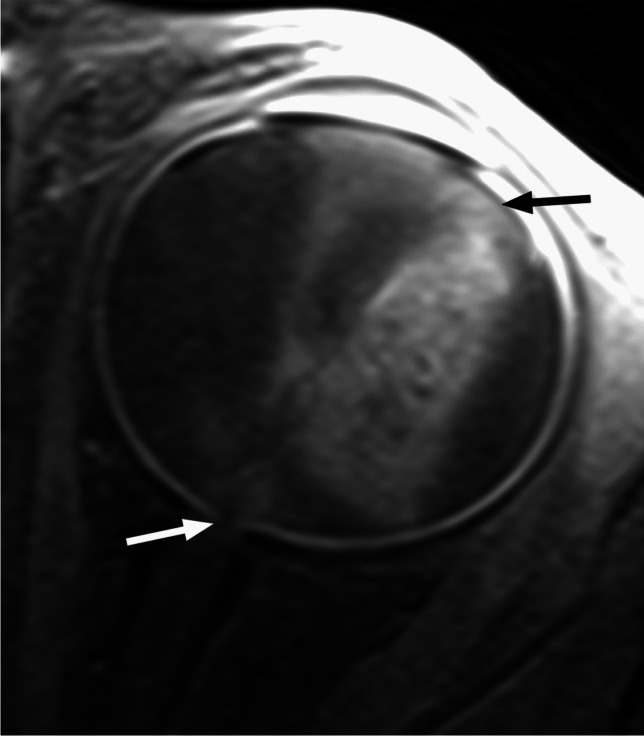
Axial postcontrast T1-weighted image in another patient with retinoblastoma shows optic nerve enhancement and interruption of the choroidal enhancement (white arrow), indicative of neoplastic invasion of the nerve. There is also extension of the enhancing mass to the anterior ocular chamber (black arrow)

**Fig. 11:**
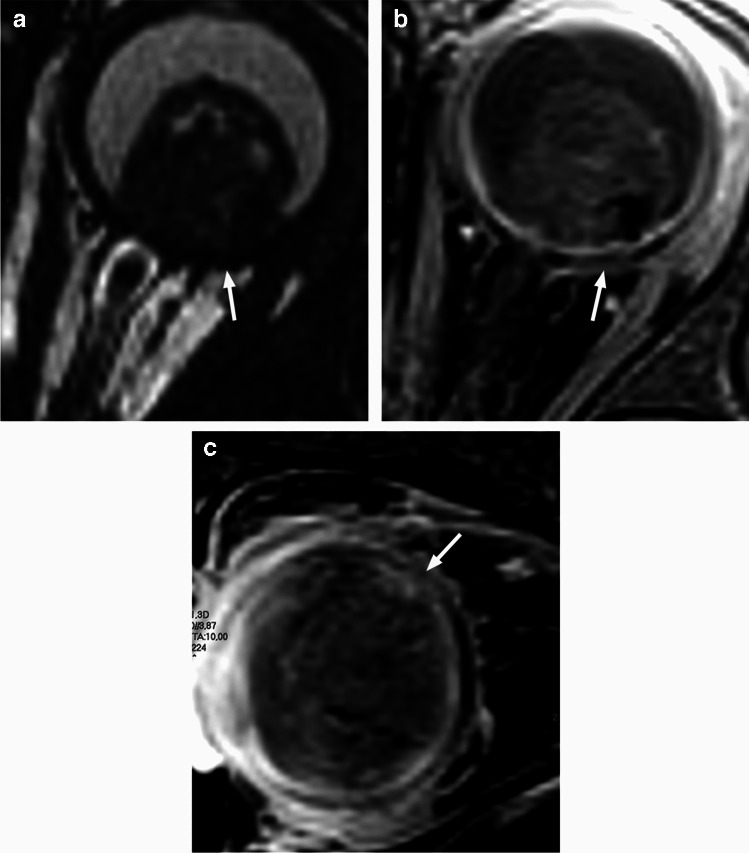
Retinoblastoma with scleral and extrascleral invasion. Axial T2-weighted image of the globe (**a**) shows a heterogenous mass with endophytic growth, consistent with retinoblastoma. The adjacent linear scleral hypointensity is irregular and discontinuous with extension of the mass into the retrobulbar fat (arrow). Corresponding postcontrast T1-weighted image (**b**) reveals enhancement of the affected sclera (arrow), which is confirmed on sagittal postcontrast T1-weighted image (**c**) demonstrating continuous enhancement of the intraocular lesion, sclera and retrobulbar fat (arrow)

Postlaminar optic nerve involvement (PLONI), choroidal invasion, vitreous seeding, anterior eye segment involvement as well as scleral and extrascleral invasion are high-risk features for metastasis and posttreatment recurrence, associated with worse outcome [8–10, 14]. Optic nerve infiltration is a route for intracranial extension, seen on MRI as abnormal post-laminar nerve thickening and enhancement [8, 9, 14] (Figs. 7 and 8). The location of the lamina on MRI is estimated at the junction between the optic disc and the midpoint between the enhancing choroid and the low intensity sclera. However, RB can cause intraorbital inflammatory response that may mimic neoplastic nerve involvement. Inflammatory nerve enhancement is equal or higher than the enhancement of the adjacent uninterrupted choroid (Fig. 9), while the invading neoplasm enhances less than the interrupted choroid, usually seen as a triangular extension of the intraocular mass [15, 16] (Fig. 10). Contrast enhancement of the anterior ocular chamber is also predictive of tumor infiltration of the optic nerve [5]. Focal discontinuity of the normal enhancement as well as focal thickening of the choroid are imaging markers of invasion. Detached small foci ‘’floating’’ within the vitreous are indicative of neoplastic seeding (Fig. 1), however the absence of these MRI findings does not rule out vitreous seeding. Infiltration of the sclera is seen as an interruption of the normally thin, linear hypointensity on both T1 and T2-weighted images and extrascleral invasion by extension of the mass into the retrobulbar fat [8, 9] (Fig. 11).

Certain molecular subtypes of RB, including a completely different mutation with normal *RB1* genes, are more aggressive and more resistant to typical therapeutic approaches. The associations between MRI features and gene expression profiles, known as radiogenomics, could provide valuable information for treatment decision-making, and there are already some promising studies [6, 10, 17]. Around 20% of children with leukocoria have lesions that simulate RB (*pseudoretinoblastoma*), most commonly *Coats disease*, followed by *persistent fetal vasculature* (PFV, previously known as persistent hyperplastic primary vitreous – PHPV) [1, 10, 18–20]. Larval granulomatosis (toxocariasis), retinal astrocytic hamartoma, familial exudative vitreoretinopathy and retinopathy of prematurity [19, 20] are substantially less common and beyond the scope of this review.

*Exudative retinitis*, commonly known as *Coats disease* is a retinal telangiectasia of unknown cause, with leakage from the dilated retinal capillaries and aneurysms resulting in accumulation of subretinal exudates and retinal detachment. The disease is almost always unilateral and affecting young boys [1, 10, 18, 20]. Shields classification divides Coats disease into 5 progressive stages and advanced stages (3–5, with retinal detachment) can mimic retinoblastoma. It has been reported that Coats disease misdiagnosed as retinoblastoma is the most common cause of inappropriate enucleation [21].

**Fig. 12:**
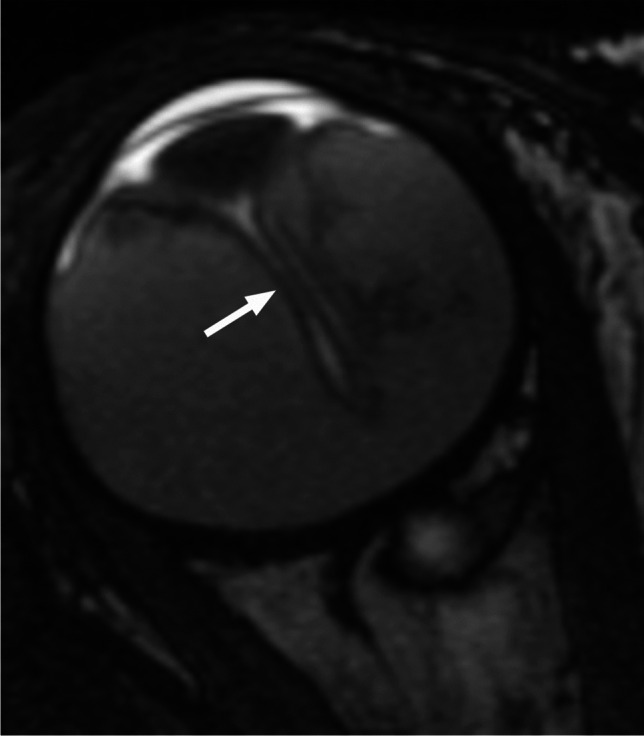
Coats disease (exudative retinitis). High resolution axial T2-weighted image of the globe reveals Y-shaped retinal detachment (arrow), without evidence of underlying mass lesion

**Fig. 13:**
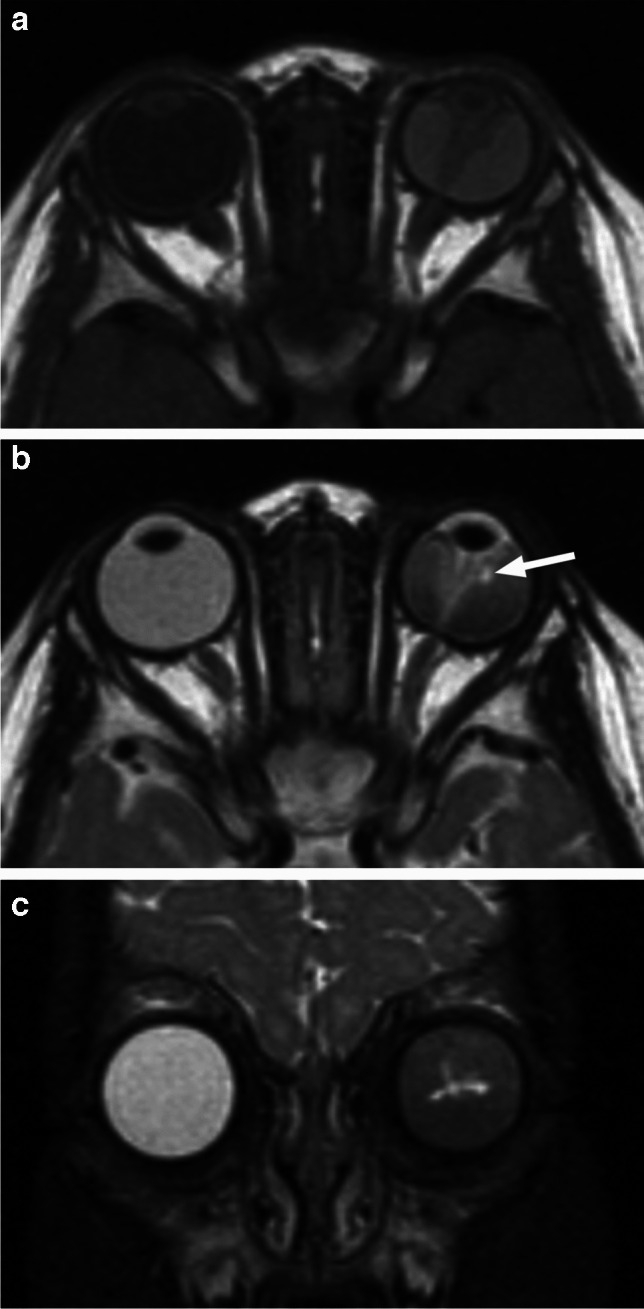
Coats disease. Axial T1-weighted image of the orbits (**a**) shows retinal detachment in the smaller left globe with hyperintensity of the exudate. Corresponding T2-weighted image (**b**) demonstrates low signal of the subretinal fluid and a retinal cyst (arrow). Extensive retinal detachment without evidence of an underlying mass in the smaller left globe is also seen on coronal STIR image (**c**)

On imaging, the affected eyes are smaller, calcifications are very rare, and solid components show only minimal to mild partial contrast enhancement. Retinal detachment is usually Y-shaped with T1 hyperintense exudate (Figs. 12 and 13), intraretinal macrocysts are frequently present (Fig. 13) and enhancement may occur outside the solid components due to contrast leakage from the abnormal vessels, creating a mismatch between enhancement and solid tissue [1, 10, 18], including subfoveal enhancing nodule [18]. ADC values of Coats disease are increased compared to the brain and significantly higher from those of retinoblastoma, allowing for separation of the two diagnoses with a high degree of accuracy [22].

The presence of a subfoveal nodule is a risk factor for the development of macular fibrosis and a worse visual prognosis, and it may become calcified in severe disease [21]. Treatment includes laser photocoagulation, cryotherapy, intravitreal anti–vascular endothelial growth factor (VEGF) therapy or corticosteroid therapy, and surgical correction of the retinal detachment. Prognosis for individuals with severe disease is poor and enucleation may be required in some cases [1, 21].

*PFV* involves a spectrum of congenital ocular abnormalities characterized by the presence of a vascular membrane behind the lens, which is caused by an incomplete in utero involution of embryological hyaloid vascular system. This results in a persistent fibrovascular stalk extending from the optic nerve to the posterior lens, connecting the retrolental membrane to the retina. The traction of this band may cause retinal detachment, retinal or vitreous hemorrhage, distortion of the ciliary body and lens, and optic nerve hypoplasia [1, 18, 20, 23].

A vast majority (90–95%) of cases are unilateral and sporadic, while bilateral presentation is commonly associated with syndromes/genetic disorders. such as trisomy 13, Walker-Warburg syndrome, and Norrie disease [1, 20, 23]. In addition to leukocoria, cataract is frequently found in the affected infants. Lensectomy with or without anterior or total vitrectomy and trabeculectomy may be performed to prevent glaucoma and preserve useful vision [1, 23].

**Fig. 14:**
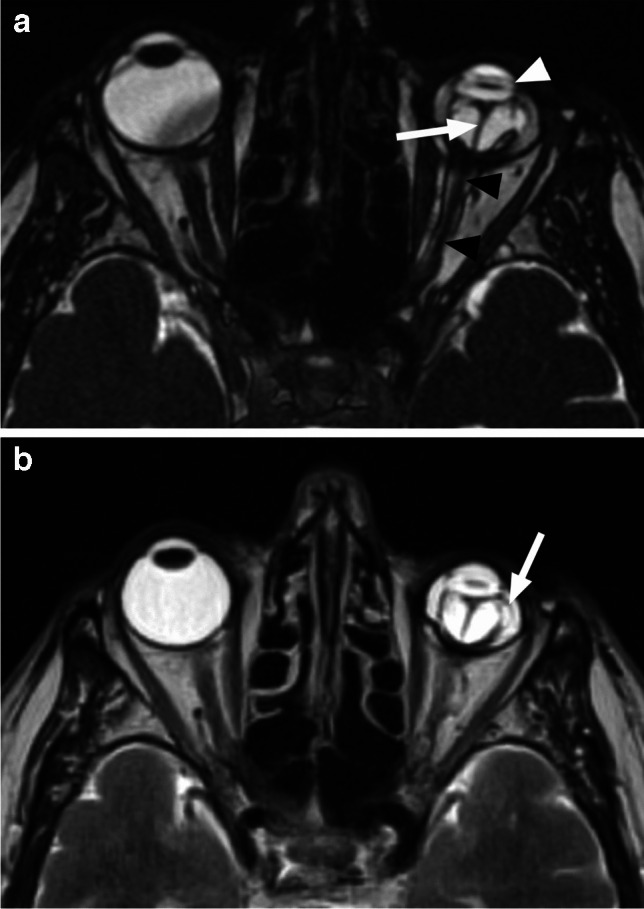
Persistent fetal vasculature (PFV). High resolution 3D T2-weighted image of the orbits in the axial plane (**a**) demonstrates microphthalmia on the left side with the characteristic central linear band (arrow) along with deformed lens and ciliary body (white arrowhead). There is also small caliber of the optic nerve (black arrowheads), consistent with nerve atrophy. Corresponding axial T2-weighted image (**b**) shows the same intraocular findings, including a retinal cyst (arrow)

**Fig. 15:**
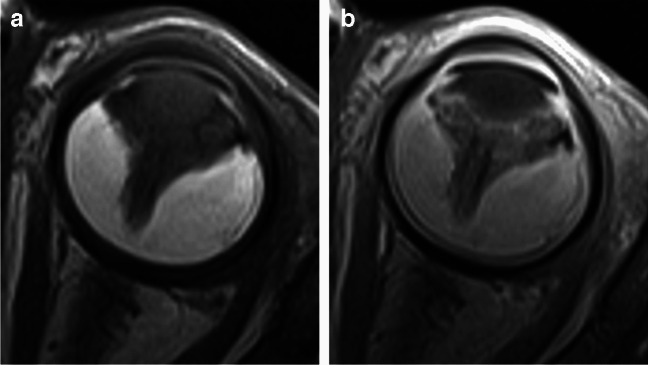
Axial T1-weighted images of the globe without (**a**) and with contrast agent (**b**) show Y-shaped retinal detachment with prominent hyperintensity of the exudate and enhancement of the cone-shaped retrolental tissue in this patient with PHV

The affected eye is almost always small, and color Doppler US reveals internal vascularity along the retrolental mass and central linear echogenic non-calcified band of tissue, due to presence of the persistent hyaloid artery [20, 23]. In addition to the characteristic morphology on imaging studies, which is often referred to as the “martini glass” sign (formed by the persistent central stalk), the lens and/or ciliary body are deformed (Fig. 14) and there is T2 hypointense contrast-enhancing cone-shaped retrolental tissue (Fig. 15). Retinal detachment with T1 hyperintensity is common, and calcification is very rare [1, 18]. SW MRI sequences may show low signal foci corresponding to persistent embryonal vessels in PFV [1]. Atrophy of the optic nerve is frequently seen (Fig. 14) and intraretinal cysts may be present [18].

A combination of larger eye size, narrow V-shaped retinal detachment and vitreous seeding strongly favors *RB*, especially with a calcified ocular mass. Smaller globe, Y-shaped retinal detachment, intraretinal cysts, and contrast enhancement outside the poorly enhancing solid component are features favoring *pseudoretinoblastoma* lesions. Optic nerve atrophy and fibrous stalk are only present in PFV, while enhancing subfoveal nodules support the diagnosis of Coats disease [18].

## Intraconal compartment

4. Optic Pathway Glioma (OPG)
5. Venolymphatic Malformations

*Optic pathway gliomas* (OPGs) are usually slow growing WHO grade 1 glial neoplasms which may involve the entire optic pathway from the optic disc, through the chiasm and hypothalamus to the optic radiations. Hereditary type is associated with neurofibromatosis type 1 (NF1) and usually involves the optic nerves, bilateral optic nerve gliomas are virtually pathognomonic for NF1. These are often low-grade pilocytic astrocytoma with an indolent course and may even spontaneously regress. The retro-orbital and intracranial optic tracts are more frequently involved in the sporadic type and sporadic OPGs typically portend a more aggressive course with worse outcomes [1, 2, 7, 24, 25]. Presentation varies from asymptomatic to proptosis, strabismus, and vision loss [24].

The diagnosis is presumed based on typical imaging features, and biopsy is usually not required. Half of the patients are diagnosed before 5 years of age, and the majority within the first decade of life.

**Fig. 16:**
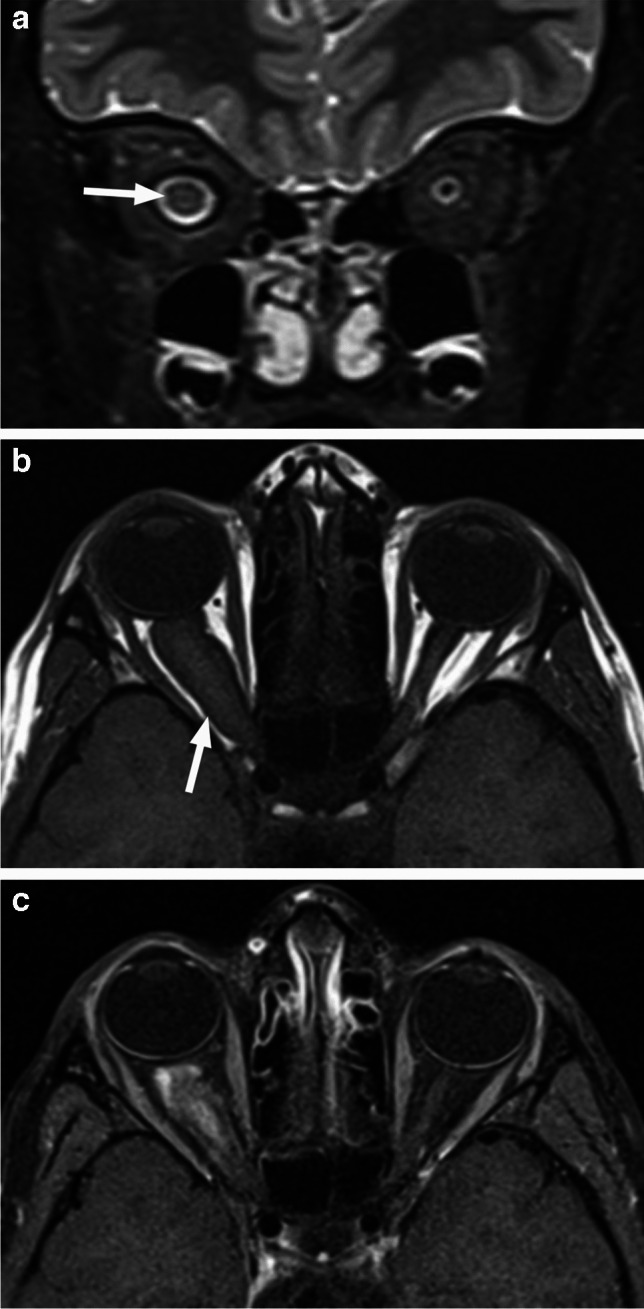
Optic nerve glioma. Coronal STIR image through the orbits (**a**) reveals a very prominent thickening and slightly increased signal intensity of the right optic nerve (arrow). Axial T1-weighted image (**b**) exhibits fusiform enlargement of the entire intraorbital nerve (arrow), which demonstrates patchy enhancement on the corresponding postcontrast T1-weighted image with fat saturation (**c**)

**Fig. 17:**
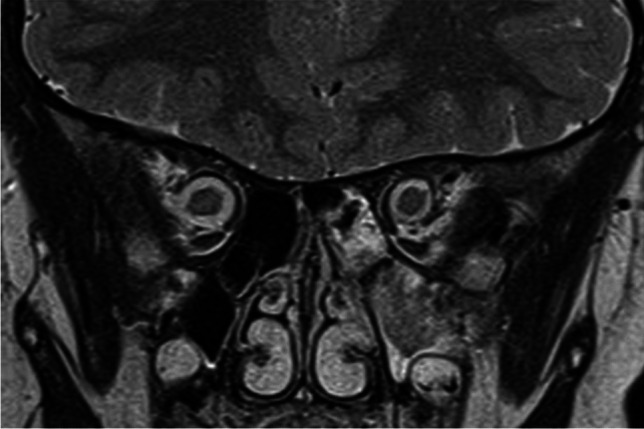
Coronal T2-weighted image shows bilaterally thickened intraorbital optic nerves, consistent with ONGs in a patient with neurofibromatosis type 1 (NF1)

**Fig. 18:**
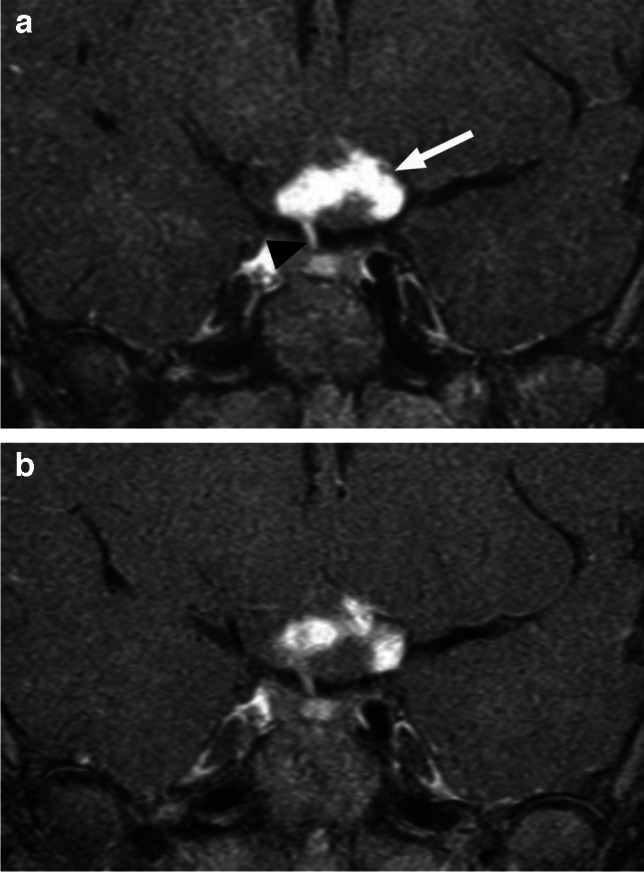
OPG in a patient with NF1. Coronal T1-weighted postcontrast image with fat saturation (**a**) shows an optic chiasm/hypothalamic mass with avid heterogenous enhancement (arrow), adjacent to pituitary infundibulum (arrowhead). Corresponding follow-up image (**b**) reveals decreased and more heterogenous enhancement, without any interval treatment

The optic nerves and/or pathways show fusiform expansion (Figs. 16 and 17), sometimes with kinking of the optic nerve and ectasia of the optic sheath. There may be enlargement of the optic canal. The lesions are of intermediate to increased T2 signal with variable contrast enhancement (including no enhancement) (Fig. 16), which can wax and wane (Fig. 18). Therefore, the assessment of interval change and/or response to treatment should be based on tumor size and extent, primarily on T2-weighted sequences, and not on alterations in enhancement [1, 2, 24].

Children with OPGs should have regular vision surveillance as almost half of them will have worsening vision or hypothalamic dysfunction. Chemotherapy is typically considered as the first-line treatment in patients with deteriorating vision, while disfiguring proptosis and large hypothalamic masses with evolving hydrocephalus may require surgical debulking [1, 24].

*Vascular malformations* are present at birth, grow proportionally with the child and do not spontaneously regress. However, they can increase in size due to hormonal changes during puberty or pregnancy or following hemorrhage, infection, inflammation, thrombosis or incomplete treatment [1, 25, 26]. Most cases are caused by sporadic gene mutations in tyrosine kinase receptor pathways responsive to vascular endothelial growth factor (VEGF). In the orbit, vascular malformations are often multicompartmental, superficial, deep or both; rarely complex involving the periorbital and intracranial compartments [26]. The deep lesions are very frequently, completely or partially, located in the intraconal compartment and my present with progressive proptosis and globe displacement. Diminished vision in the affected eye is primarily due to exposure keratopathy and compressive optic neuropathy [25].

**Fig. 19:**
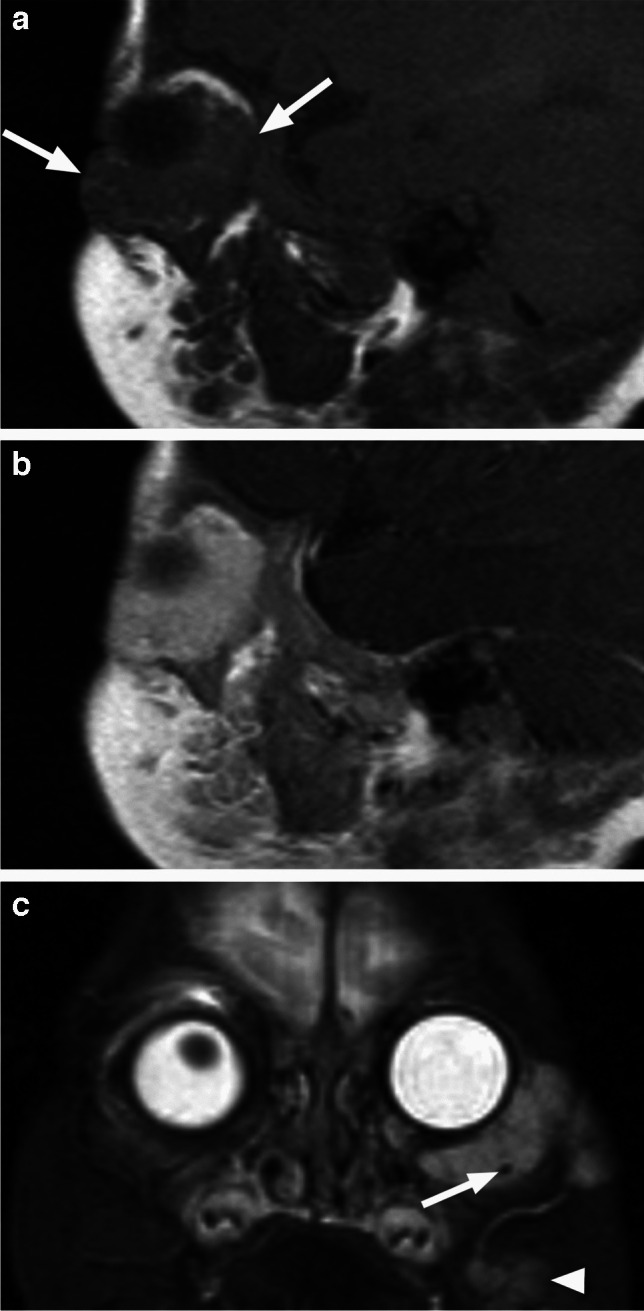
Venous malformation. Sagittal T1-weighted image (**a**) shows an isointense preseptal and postseptal intraorbital lesion (arrows), which is enhancing on the corresponding delayed postcontrast image (**b**). Coronal STIR image (**c**) demonstrates increased signal of the lobulated mass with an internal oval hypointensity, indicative of a phlebolith (arrow), without flow voids. The irregular trans-spatial lesion is contiguous with an additional facial component (arrowhead), which is also present on the sagittal images

V*enous malformations* (VMs) are the most common low-flow vascular malformations, composed of an abnormal venous network of varying size that is embedded in fibrous tissue. The lesions are lobulated and infiltrating with ill-defined margins, frequently crossing anatomic boundaries and involving both the pre- and post-septal tissues of the orbit (Fig. 19). Phleboliths are essentially pathognomonic for VMs and are present in about 50% of the lesions, seen on CT as ovoid calcifications within dilated venous channels or a focal loss of signal within a T2 hyperintense lesion on MRI. VMs are T2 hyperintense without flow voids, showing early patchy contrast enhancement with slow gradual filling [26, 27]. Superficial VMs are compressible lesions on ultrasound with low and slow flow which intensifies with the Valsalva maneuver.

**Fig. 20:**
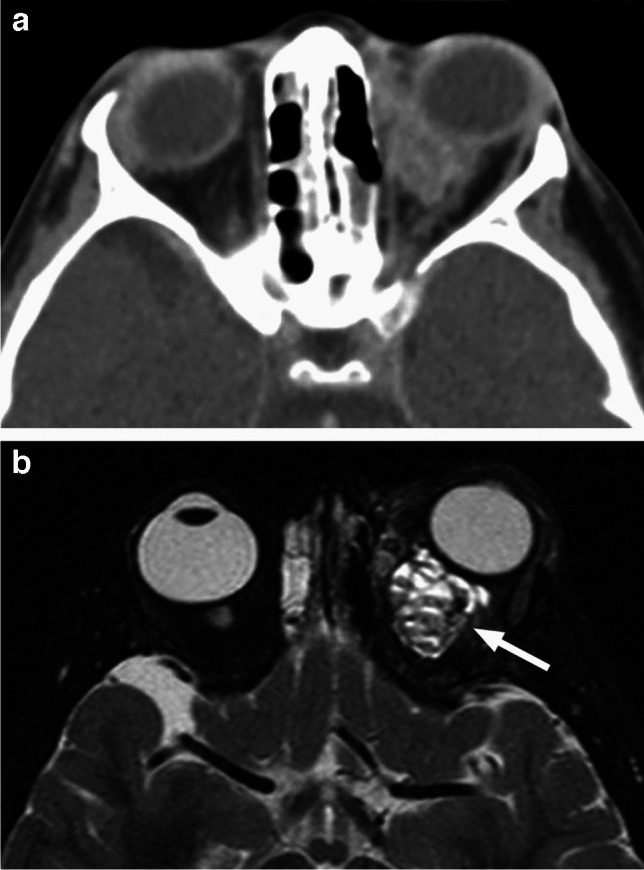
Lymphatic malformation. Axial postcontrast CT image (**a**) shows a heterogenous predominantly intraconal left orbital mass leading to left ocular proptosis. Corresponding T2-weighted image with fat saturation (**b**) reveals multicystic structure of the lesion with characteristic layering fluid levels (arrow)

*Lymphatic malformations* (LMs, *previously with the misnomer lymphangiomas*) consist of dilated lymphatic channels separated by thin septa, without vascularization. Conglomerates of lymphatic chambers (which may be subdivided into macrocystic, microcystic and mixed) are seen as T2 hyperintense fluid-filled spaces with possible thin peripheral enhancement and T2 dark fibrous septa, without any solid enhancing component. The signal intensity of these cysts varies depending on the amount of proteinaceous content and hemorrhage, usually iso to slightly hyperintense on T1-weighted sequences. Fluid-fluid levels within the dilated lymphatic channels are virtually pathognomonic for LMs, usually indicating recent intralesional bleeding [26] (Fig. 20).

**Fig. 21:**
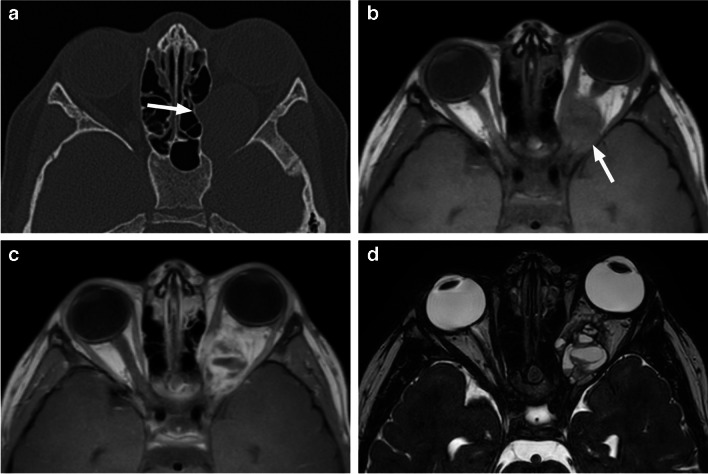
Axial CT image with bone algorithm and window (**a**) demonstrates mild expansion and smooth remodeling of the bony orbital walls on the left side with most prominent medial deviation of the lamina papyracea (arrow). There appears to be an intraconal mass lesion. Corresponding T1-weighted image (**b**) reveals a slightly heterogenous predominantly isointense intraconal mass (arrow), which exhibits partial patchy enhancement on the postcontrast image (**c**). High resolution 3D T2-weighted image (**d**) at the same level shows the characteristic fluid-fluid levels within the multicystic portion of this venolymphatic malformation (VLM)

A trans-spatial orbital multicystic lesion with a non-enhancing portion containing fluid levels and a slowly enhancing bright T2 solid component is consistent with *venolymphatic malformation* (VLM), which is a combination of venous and lymphatic structures featuring imaging characteristics of both VM and LM [1, 7, 25] (Fig. 21). Vascular malformations may lead to smooth remodeling of the adjacent orbital walls. A strong association with intracranial vascular anomalies has been reported in patients with extensive orbital VLMs [28].

Percutaneous sclerotherapy is a first-line therapy for *VLMs* with negligible recurrence rates and improvement in vision and proptosis after treatment. Systemic corticosteroids may be used for alleviation of acute symptoms from mass effect, and a substantial decrease in size can be achieved by medical therapy with sirolimus [1, 25, 27].

Optic nerve sheath meningioma and orbital Schwannomas are very rare in children, more frequently seen in in the context of neurofibromatosis type 2 [1, 7]. Their imaging features are the same as in the adult patients (such as avid peripheral nerve sheath enhancement and tram-track calcifications of meningioma) and will not be further discussed.

## Extraconal compartment

1. Rhabdomyosarcoma (RMS)
2. Hemangiomas
3. Lymphomas
4. Neurofibromas

*Rhabdomyosarcoma* (RMS) is the most common mesenchymal neoplasm and extraocular malignancy in the pediatric orbit with a mean age of 6–8 years at diagnosis, presenting as rapidly progressive unilateral proptosis. The most common subtype is embryonal RMS, which typically occurs in the superior medial (superonasal) aspect of the orbit. The more aggressive, less common alveolar type has a predilection for the inferior orbit and is usually seen in adolescents [7, 25, 29]. RMS has the propensity to arise from the extraocular muscles and the eyelid, but it may also involve the intraconal compartment and rarely invade the surrounding bone and cranial nerves. Invasion of the bony walls or the optic nerve classifies orbital RMS as a high-risk parameningeal location [1, 30].

**Fig. 22:**
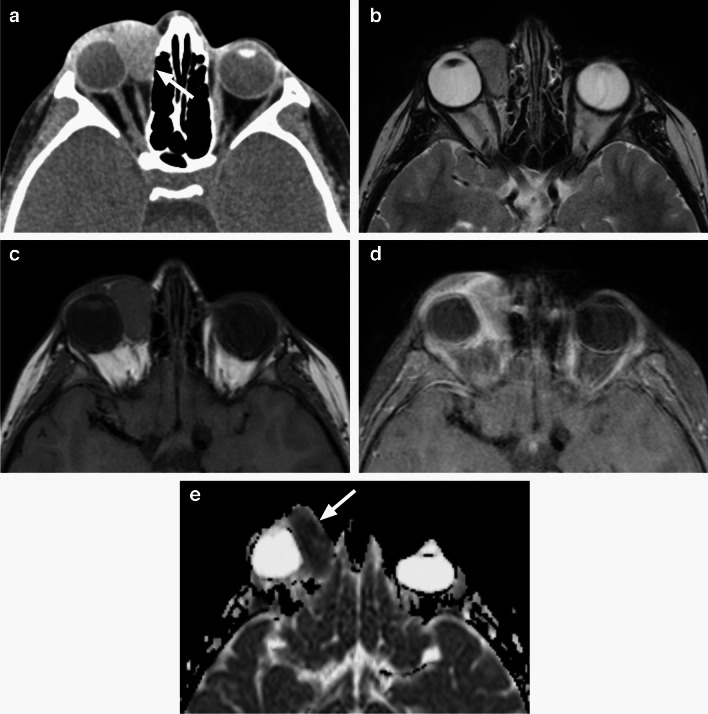
Rhabdomyosarcoma. Axial non-enhanced CT image (**a**) shows a well-defined homogenous oval extraconal lesion (arrow) in the superonasal aspect of the right orbit, isodense with extra-ocular muscles and mildly compressing the globe. Adjacent medial orbital wall appears intact. Axial T2-weighted (**b**) and T1-weighted (**c**) images at a similar level show homogenous intermediate to slightly increased signal intensity of the mass, without flow voids. There is mildly heterogenous enhancement on axial postcontrast T1-weighted image with fat saturation (**d**, suboptimal image quality due to motion artifacts) and low diffusivity of the mass (arrow), which is hypointense to the brain on corresponding ADC map (**e**)

*RMS* is most commonly a well circumscribed extraconal soft tissue mass with displacement and compression of the globe, extra-ocular muscles and the optic nerve. MRI shows low ADC values and contrast enhancement of the lesion, which is predominantly isodense/isointense to the muscles on CT and T1-weighted sequences, with variable, mostly intermediate to increased T2 signal intensity [1–3] (Fig. 22). T2 signal and enhancement pattern tend to be more heterogeneous in larger lesions due to necrosis and hemorrhage. CT is complementary to MRI for evaluation of possible bone invasion (which then classifies as parameningeal RMS) [3, 30]. On imaging studies RMS could be mistaken for a benign lesion, primarily infantile hemangioma, as it frequently has well-defined margins and may even contain serpentine flow voids [24, 29]; the key distinguishing feature is the low diffusivity on ADC maps, hypo to isointense compared to the brain [1, 31, 32] (Fig. 22).

Treatment includes resection and chemotherapy. The prognosis is excellent, with 5-year survival rates of approximately 95% [1]. In up to one-third of cases, a substantial residual mass is found following chemotherapy and radiation, which may be associated with new bone erosions, but contains only mature rhabdomyoblasts without malignant neoplastic tissue. Increased ADC values and reduced FDG uptake compared to the initial mass help in differentiation from residual RMS [33, 34].

*Infantile hemangiomas* (IH) are the most common benign vascular neoplasms in infancy, which are usually not present at birth but are typically noticed within the first few weeks of infancy. These rapidly growing lesions go through a rapid proliferative phase in the first year of life, followed by the prolonged involutionary phase for up to 10 years and regression until late childhood [1, 25, 26, 29]. Most IHs arise in the head & neck, especially the orbit, where they are usually located anteriorly - in the preseptal soft tissue and superomedial extraconal space (like rhabdomyosarcomas) but may involve multiple contiguous compartments. They are glucose transporter protein isoform–1 positive (GLUT-1, which is helpful in differentiating vascular neoplasms from malformations in histological specimens) densely vascular lesions, formed by proliferating lobules of endothelial cells. The diagnosis is clinical, based on the raspberry-red cutaneous stains of increasing size with a sharp margin [26, 35]. Imaging is rarely required for correct diagnosis and treatment is frequently not needed; however, both are necessary for any significant threat to vision (due to prolonged eyelid closure, strabismus, astigmatism, and stretching of the optic nerve). Propranolol is a highly effective standard therapy (with response rate of over 98%), while surgical approaches, either open or laser, possibly with preoperative embolization and other medications, are reserved for select circumstances [1, 35].

**Fig. 23:**
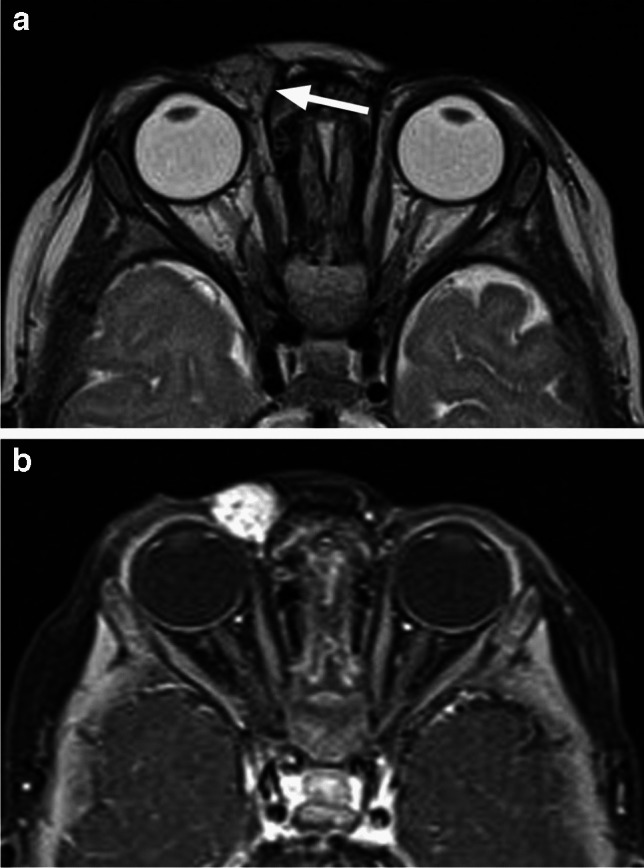
Infantile hemangioma. Axial T2-weighted image through the orbits (**a**) shows a small preseptal and superomedial extraconal mass (arrow) with internal dark flow voids and fibrous septa. Corresponding postcontrast T1-weighted image with fat saturation (**b**) demonstrates avid dense enhancement of the well-defined lesion

**Fig. 24:**
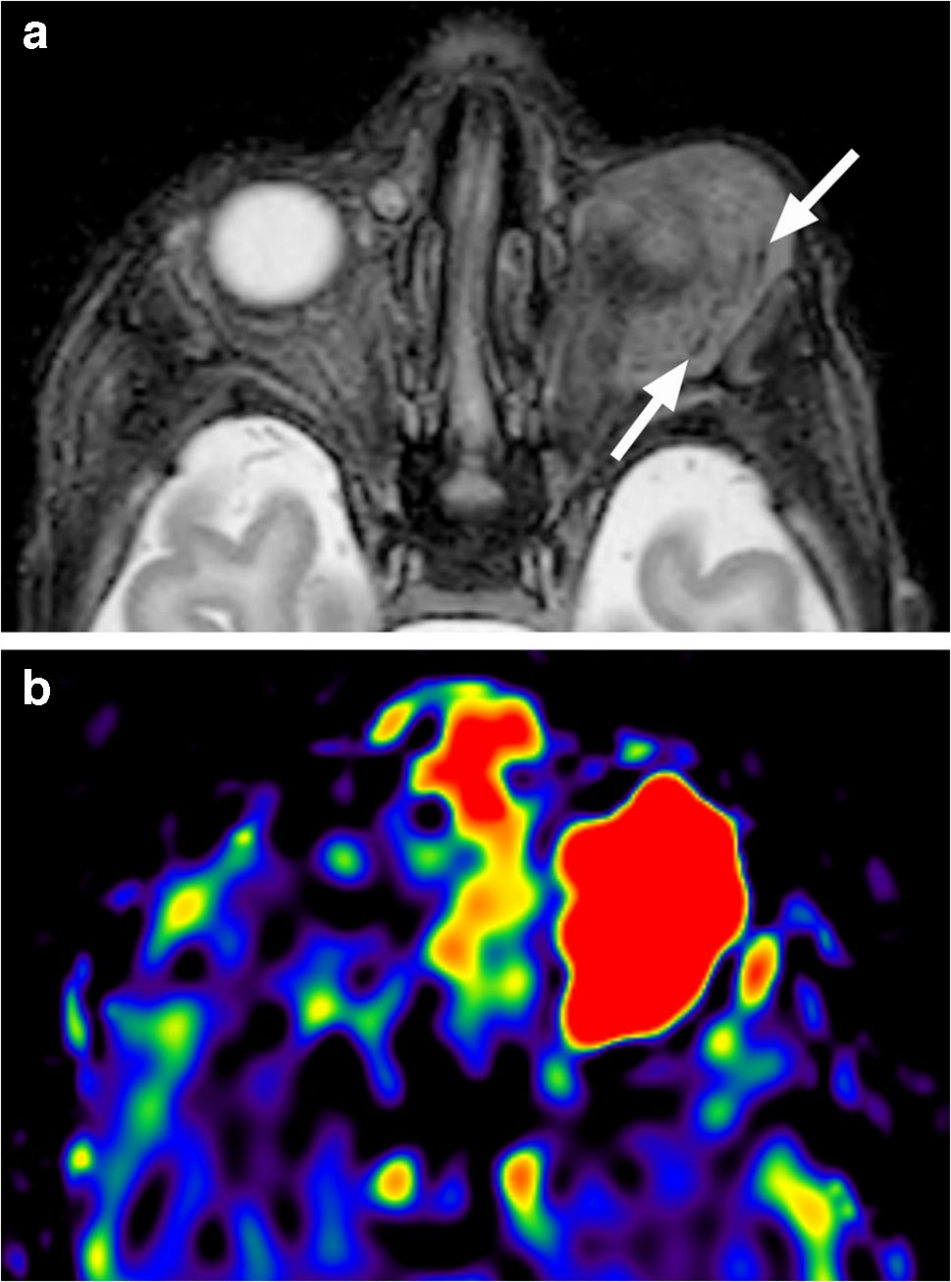
Axial T2-weighted image of the orbits (**a**) shows a preseptal and postseptal intraorbital lesion containing flow voids (arrows), which demonstrates intense homogenous hyperperfusion with arterial spin labeling (ASL) (**b**). The lesion also demonstrated prominent homogenous enhancement and high ADC values, hyperintense to the brain (not shown), consistent with infantile hemangioma

Ultrasound is the imaging modality of choice, especially for the superficial lesions, offering good assessment of the lesion architecture, vascularity and flow dynamics [26, 35]. MRI is performed prior to treatment and is also indicated for evaluation of possible intracranial extension and secondary findings suggestive of specific syndromes (primarily PHACE - Posterior fossa malformations, Hemangiomas, Arterial anomalies, Coarctation of the aorta, Eye anomalies), in children with associated segmental (facial or scalp) infantile hemangiomas [1, 26, 35]. Orbital IHs are well-defined, lobulated masses with high T2 signal, internal dark flow voids and fibrous septa between the lobules (Fig. 23). They show high ADC values and avid, homogeneous contrast enhancement, along with intense homogenous hyperperfusion with arterial spin labeling (ASL) [2, 36] (Figs. 23 and 24). In the involutionary phase the T1 signal intensity increases while enhancement and flow voids diminish as the vessels are replaced by fat and fibrotic tissue [25, 26, 35].

Benign vascular neoplasms can be mistaken for vascular malformations, but even more frequently vascular malformations are misdiagnosed as vascular tumors, most commonly infantile hemangiomas. Inappropriate misnomers and false classification are responsible for wrong treatment approaches, which may delay appropriate therapy, or lead to significant morbidity and mortality [35]. Venous malformations also show increased T2 signal, however without flow-voids and with gradual enhancement; they are irregular and ill-defined lesions, frequently contain phleboliths and may be trans-spatial. Infantile hemangiomas can be differentiated from RMS on MRI by their high ADC values.

**Fig. 25:**
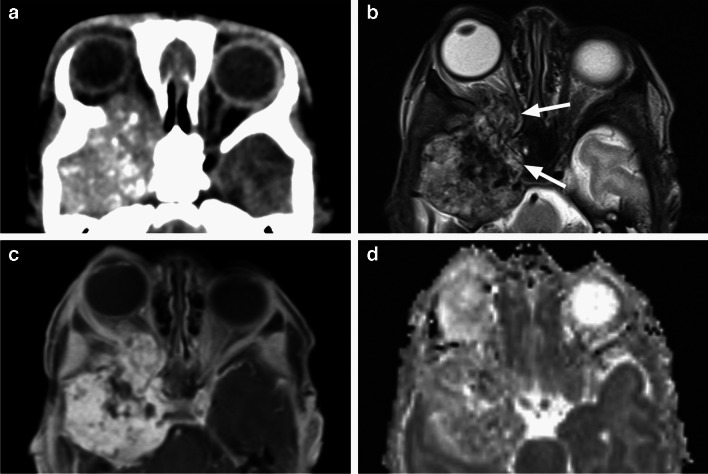
Congenital hemangioma. Axial CT image (**a**) in a neonate with prominent proptosis shows a heterogeneous right intraorbital and intracranial mass containing calcifications. Follow-up MRI scan with axial T2-weighted image at a similar level (**b**) reveals flow voids (arrows) within the heterogenous mass (arrows). Corresponding postcontrast T1-weighted image (**c**) shows intense enhancement of the lesion, while ADC map (**d**) demonstrates increased diffusivity (hyperintensity compared to the brain)

*Congenital hemangiomas* (CH) are rare in the orbit, clinically apparent at birth, showing essentially no further growth and lacking immunological marker GLUT-1. CH may show rapid involution (RICH type), no involution (NICH type) or partial involution (PICH type) [24, 26, 35] and cannot be reliably distinguished from IH on imaging alone. They sometimes contain calcifications and may be more heterogeneous on T2-weighted images along with a patchy contrast enhancement [24] (Fig. 25).

*Lymphoma* of the orbit can occur primarily or may be a secondary manifestation of systemic lymphoma in older children, usually non-Hodgkin lymphoma (NHL), and patients who present with primary orbital lymphoma may eventually develop systemic NHL [7]. It commonly involves ocular adnexal structures, such as the conjunctiva, lacrimal apparatus, eyelid, and/or extraocular muscles [1]. The typical presentation is painless proptosis [7]. Extranodal marginal zone lymphoma is the most common subtype and has an excellent prognosis with low-dose radiation therapy. Chemotherapy is the second-line treatment [1].

Orbital lymphoma is typically nodular, involving the superolateral quadrant and lacrimal gland, or it may be infiltrative with intraconal extension and encasement of the posterior globe. Bilateral lesions raise the suspicion for lymphoma. On MRI, the lesions are characteristically homogenous with very low ADC values and moderate to marked contrast enhancement. T1 and T2 signal intensity are variable, usually slightly T2 hypointense and T1 hyperintense. Lymphoma also shows increased perfusion and high metabolic activity on FDG PET (combined with CT or MRI) [1], and is homogenously hyperdense on non-enhanced CT, typically without erosion of the adjacent bone.

Idiopathic orbital inflammation (IOI, also known as orbital pseudotumor) has overlapping imaging features with lymphoma, but substantially different treatment, and, in most cases, distinctive clinical presentation with painful swelling. Both ASL perfusion and diffusion MR imaging are useful to differentiate the two entities, with lymphoma having relatively higher perfusion and lower ADC values [1, 37]. ASL and especially the combination of ASL and ADC values are very useful and valuable tools that have been found to improve the diagnostic confidence for pediatric orbital lesions [38].

*Plexiform neurofibromas* are peripheral nerve sheath tumors that occur almost exclusively in children with Neurofibromatosis type I (von Recklinghausen disease; NF-1) and often manifest by early childhood [1]. Neurofibromas are a benign proliferation of peripheral neural elements, with the plexiform variety differing from focal neurofibromas in that they arise from multiple nerve fascicles [2]. These lesions are common in the head and neck, particularly in the periorbital region and are most appropriately labeled as orbital-periorbital plexiform neurofibromas (OPPNs) [1, 39]. Blepharoptosis is the most common and notable presenting sign of OPPN with an incidence of nearly 100%, followed by proptosis, eyelid edema and strabismus. Most OPPNs track along the distribution of the trigeminal nerve, frequently arising in the superior extraconal compartment from the ophthalmic (V1) nerve branches. Management depends on the extent and degree of visual impairment and can include observation with surveillance imaging and surgical debulking. Early medical treatment with targeted biologic agents may prevent progression and the resultant facial disfigurement [38]. Possible transformation to a highly aggressive malignant peripheral nerve sheath tumor occurs mostly in adults and the risk is exceedingly rare for *OPPN* [1, 39].

**Fig. 26:**
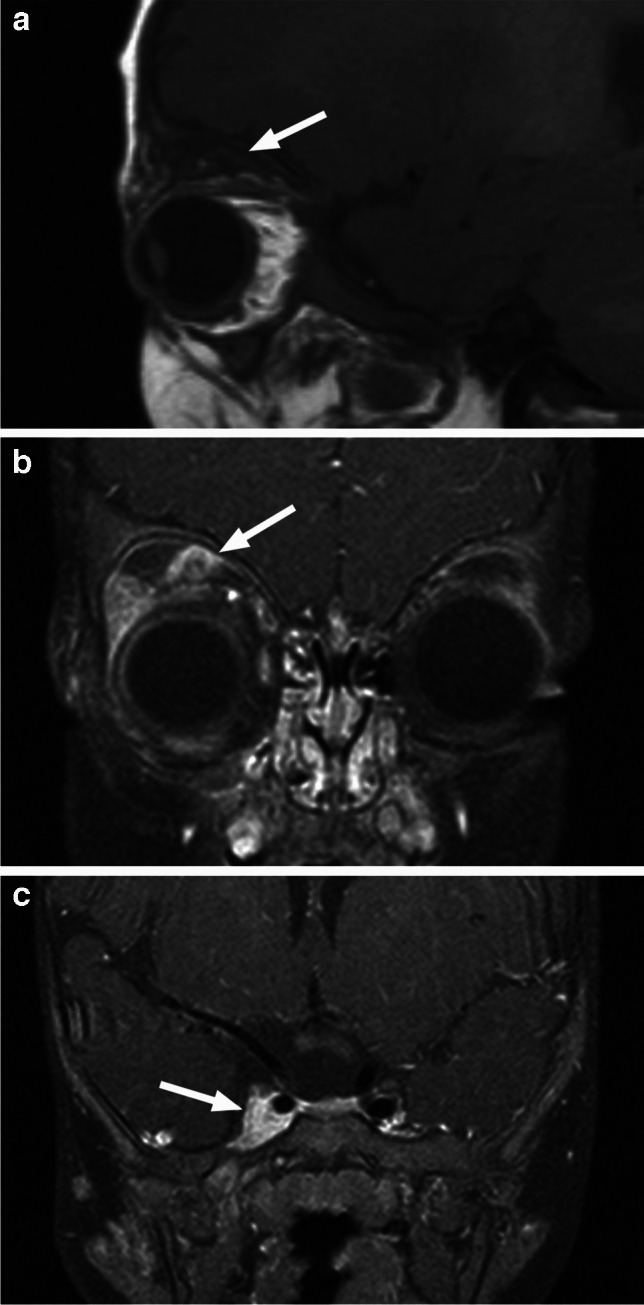
Plexiform neurofibroma. Sagittal T1-weighted image (**a**) shows a heterogenous lesion (arrow) in the superior extraconal compartment. There is heterogenous enhancement of the irregular mass (arrow) in the frontal nerve location and mild expansion of the orbit on postcontrast coronal T1-weighted image with fat saturation (**b**). A more posterior coronal image (**c**) shows extension of the lesion into the enlarged right cavernous sinus (arrow)

MRI scan of the brain and orbits should be performed in all children with a suspected OPPN [1, 39]. OPPN is a lobulated lesion often resembling a conglomerate of wormlike or grapelike masses, showing nonspecific usually decreased T1 and slightly increased T2 signal intensity with variable, often mild and heterogenous, contrast enhancement pattern (Fig. 26). A distinctive finding is a hypointense center (“target sign”), which may be seen on T2 -weighted images [1, 2, 24]. The lesion is commonly found along the frontal nerve, and it may enlarge the orbit with widening of the superior orbital fissure and extend into the cavernous sinus (Fig. 26). Other features of NF-1 may also be present, including foci of abnormal signal intensity (FASI), intracranial vasculopathies, sphenoid wing dysplasia, and optic pathway gliomas [24, 39].

Teratoma (showing characteristic fat density and calcifications, along with heterogenous enhancement), infantile fibromatosis, (infantile) fibrosarcoma and venous varix [1, 24, 29] are beyond the scope of this review and will not be discussed.

## Orbital walls

1. Neuroblastoma
2. Leukemia/Myeloid sarcoma
3. Langerhans cell Histiocytosis (LCH)
4. Dermoid

*Neuroblastoma* is the most common extracranial solid childhood malignancy that can occur anywhere along the sympathetic nervous system, arising from pathologic neural crest cell derivatives, most commonly from the adrenal glands. It is also the most common primary pediatric malignancy to metastasize to the orbit, which happens in approximately 25% of cases [25, 40]. More importantly, around 30% of patients have orbital metastases at diagnosis and it is frequently the first clinical sign of disease, preceding evidence of the primary neoplasm [2, 40].

It is postulated that tissues derived from the neural crest may provide the appropriate “soil” to support metastasizing neuroblastoma, possibly explaining its remarkable tendency to involve the periorbital calvarium, which is also derived from the neural crest [40]. Alternatively, the orbital lesions may possibly represent synchronous neoplasms, rather than metastatic ones. The only currently established example of such mechanism with separate independent neoplasms is in children with germline *RB1* gene mutations, who are at increased risk for bilateral and trilateral retinoblastoma; however, at least a few cases of neuroblastoma presenting as multifocal synchronous lesions have been reported [41, 42].

Most patients are less than 2 years old and the initial clinical manifestation is typically periorbital ecchymosis (“raccoon eyes”) with proptosis, which may potentially be mistaken for (non-accidental) trauma [1, 40]. Treatment is multimodal including chemotherapy, resection, hematopoietic stem cell rescue and radiation therapy [2, 25].

**Fig. 27:**
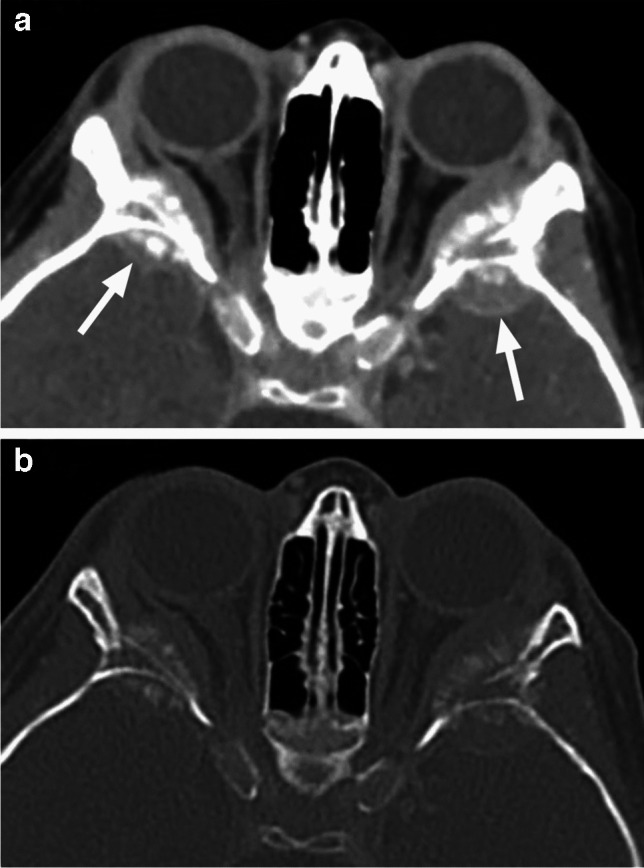
Axial postcontrast CT in soft tissue (**a**) and bone (**b**) window shows bilateral destructive lesions of the sphenoid wings/dorsolateral orbital walls with associated soft tissue components (arrows) and spiculated periosteal reaction in this patient with neuroblastoma metastases

**Fig. 28:**
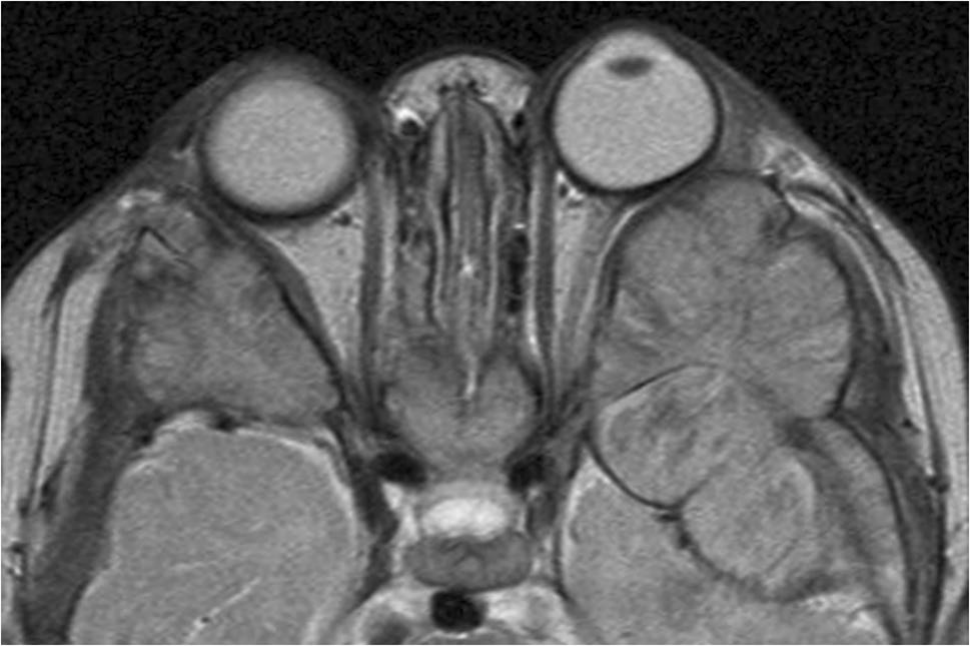
Neuroblastoma metastases. Axial T2-weighted image shows left greater than right expansile masses centered at sphenoid wings with displacement of the globe and intraorbital contents. Striated appearance of the lesions corresponds to the spiculated periosteal reaction

As with other orbital wall lesions, CT is very helpful for detection and characterization of the bony changes. Osseous metastases/synchronous primary neuroblastoma lesions are expansile with new bone formation. The spiculated ‘’hair on end’’ periosteal reaction is best seen on bone algorithm and window CT images, characteristically arising from the posterolateral orbital walls, where the frontal bone and greater wing of the sphenoid meet (Fig. 27). MRI shows mild T1 hypointensity and intermediate to slightly hyperintense T2 signal with very low ADC values and avid contrast enhancement [1, 25, 40] (Fig. 28). Bilateral orbital involvement is present in about half of cases [40, 43]. Most neuroblastomas are detected by MIBG bone scan and FDG PET (with CT or MRI) provides complementary information in other cases [1].

Leukemia is the most common pediatric malignancy with acute lymphoblastic leukemia (ALL) accounting for 80% and acute myeloid leukemia (AML) for 20% of cases [25, 43]. Extramedullary accumulation of leukemic cells in bone and soft tissue is called *myeloid sarcoma* (MS) or *granulocytic sarcoma* (previously termed *chloroma*) and can be an orbital manifestation in children with acute myeloid leukemia (AML), typically arising in the subperiosteal region of the osseous walls. MS may occur in a patient with a known AML, possibly heralding relapse; may be the presenting sign of coincident systemic disease; or may antedate the development of systemic disease by months or even years. The mean age of diagnosis is around 8 years, and the most common clinical presentation is rapidly progressing proptosis [25, 43, 44]. It is important to make an accurate, timely diagnosis in cases where orbital MS appears before systemic disease, as starting systemic chemotherapy early has a dramatic effect on prognosis [44].

**Fig. 29:**
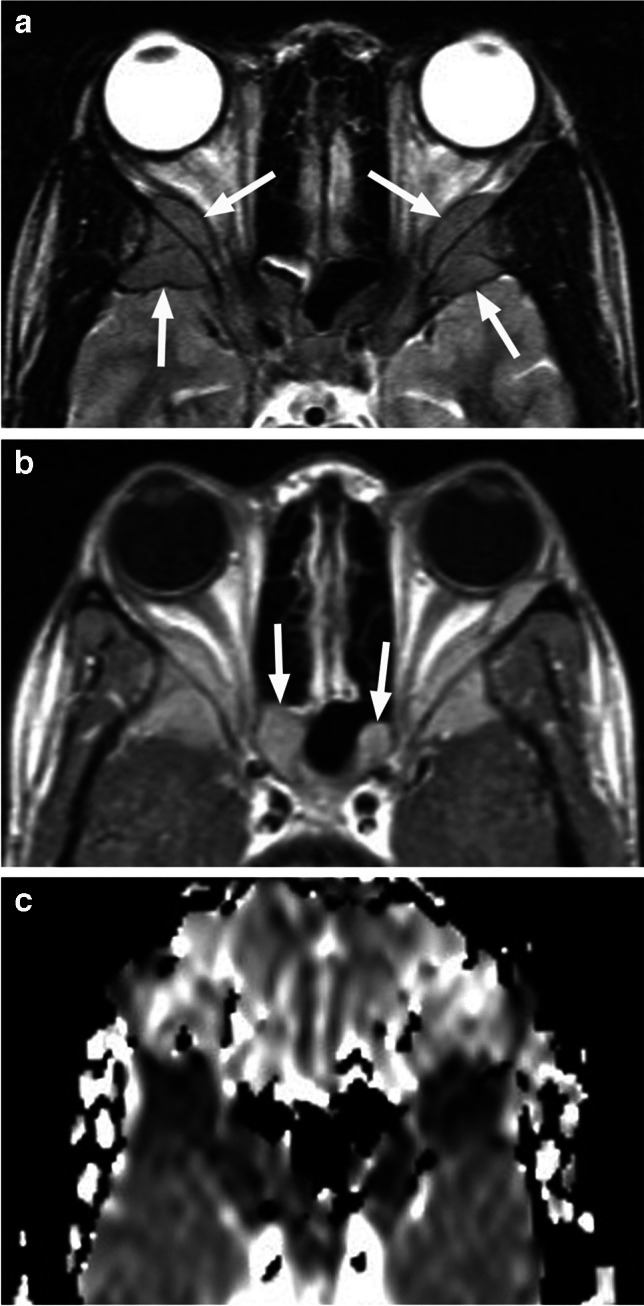
Axial T2-weighted image of the orbits (**a**) shows bilateral homogenous mildly hypointense masses (arrows) centered at the posterior lateral orbital walls/sphenoid wings. There are preserved central hypointense lines in the cortical bone location. Corresponding postcontrast T1-weighted image (**b**) demonstrates mild homogenous enhancement of the lesions, which also extend into the sphenoid sinuses (arrows). Axial ADC map at a similar level (**c**) reveals dark signal of the masses, representing very low diffusivity. Acute myeloid leukemia (AML) was diagnosed in this patient presenting with orbital myeloid sarcoma

**Fig. 30:**
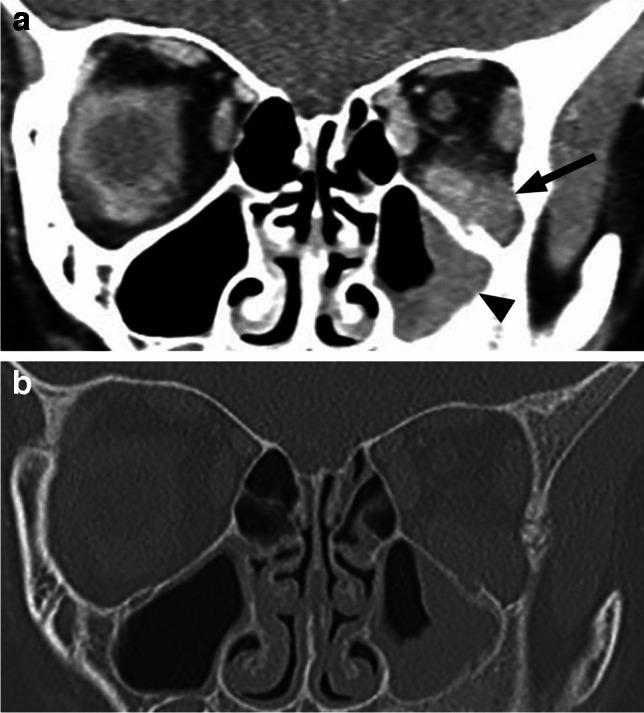
Orbital myeloid sarcoma. Coronal postcontrast CT in soft tissue (**a**) and bone (**b**) window and algorithm shows an infiltrative soft tissue mass (arrow) in the inferior left orbit extending to the left maxillary sinus (arrowhead) without bone erosion in this patient with AML

**Fig. 31:**
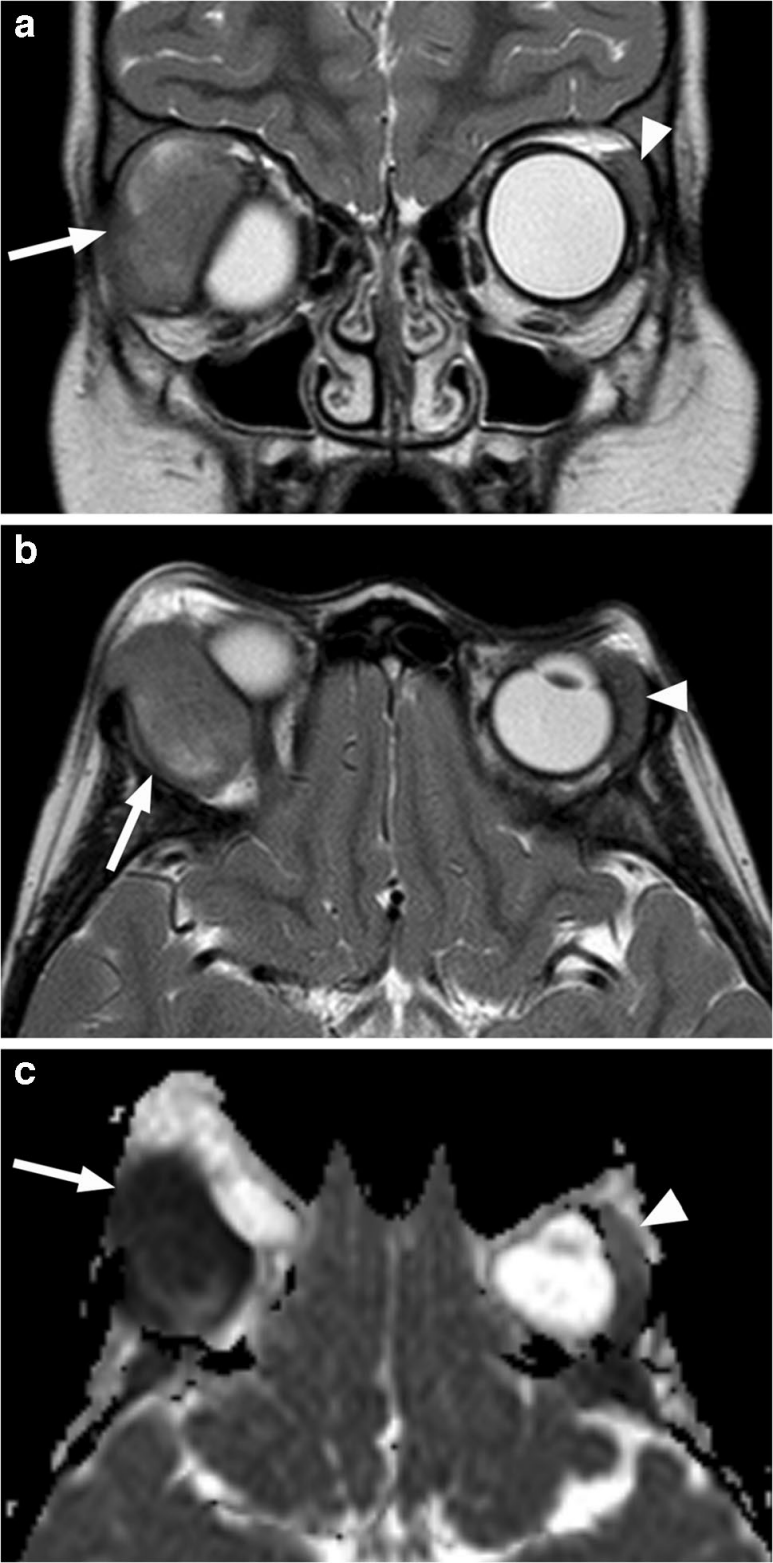
Coronal (**a**) and axial (**b**) T2-weighted images as well as axial ADC map (**c**) demonstrate an extraconal slightly heterogeneous mass (arrows) in the superolateral right orbit, likely arising from the lacrimal gland, with compression of the globe. Compared to the normal gland on the left (arrowheads), the lesion shows slightly increased T2 signal and decreased, very low ADC values (hypointense to the brain). The imaging appearances in this patient with acute lymphoblastic leukemia (ALL) are typical for orbital lymphoma, reflecting the similarity of these two mass lesions

Similar to neuroblastoma, myeloid sarcoma has the predilection for the lateral orbital walls, and it may also be bilateral, however it is encasing rather than invading the normal structures [43]. Similar to lymphoma, the masses are homogeneous and slightly hyperdense on CT with low T1 and intermediate to low T2 signal intensity, along with low ADC values and homogeneous contrast enhancement (Fig. 29). There is typically no destruction of the infiltrated bone (Fig. 30), which is however involved and shows diffuse T1 hypointensity (loss of normal high T1 signal of the bone marrow) [1]. Leukemia may also arise from and involve other intraorbital structures, such as the extraocular muscles or the lacrimal gland, with the imaging appearance closely resembling lymphoma [43, 45, 46], and this may be a feature of very rare orbital manifestations of ALL (Fig. 31).

*Langerhans cell histiocytosis* (LCH) results from proliferation of the Langerhans cell, an immature dendritic cell of bone marrow origin and is considered a neoplastic disease. The clinical spectrum of LCH ranges from a single lesion to a multisystem disease [1, 47]. Erythema, upper eyelid edema, periorbital swelling, pain, and proptosis are possible manifestations of orbital involvement [1, 47], however orbital lesions can be clinically silent and incidentally detected at imaging performed for initial systemic assessment [47]. Single-site disease is associated with the best prognosis and may resolve spontaneously (following biopsy) but is usually treated with curettage and intralesional steroids. Multifocal disease is treated with chemotherapy [48].

**Fig. 32:**
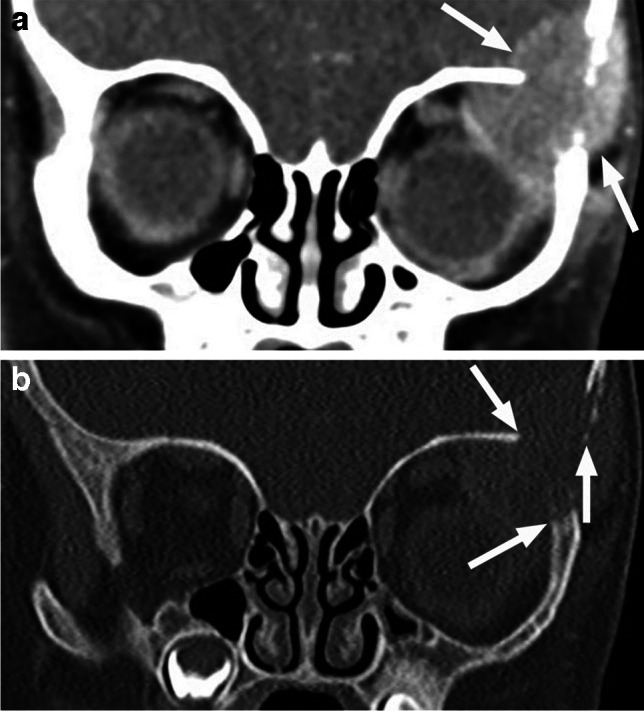
Coronal postcontrast CT images in soft tissue (**a**) and bone (**b**) window and algorithm show a destructive lesion of the left superolateral orbital wall with soft tissue components exhibiting slightly more intense peripheral enhancement (arrows in **a**) and relatively well-defined bony margins (arrows in **b**) in this patient with Langerhans cell Histiocytosis (LCH)

**Fig. 33:**
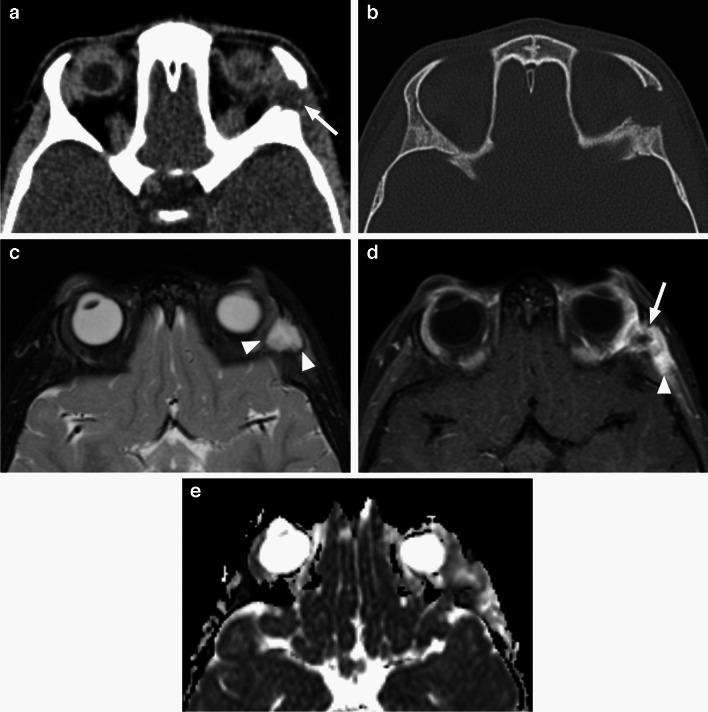
LCH. Axial nonenhanced CT image in soft tissue window and algorithm (**a**) shows a slightly hypodense osteolytic lesion (arrow) of the left superolateral orbital wall. Axial CT image at a similar level in bone window and algorithm (**b**) reveals smooth sharp borders of the destructed bone. Corresponding T2-weighted image (**c**) shows hyperintensity and a thin dark rim (arrowheads) of the lesion. There is predominantly peripheral enhancement (arrow), which extends into the surrounding tissues (arrowhead) on axial postcontrast T1-weighted image with fat saturation (**d**). The lesion shows centrally high and peripherally mild hyperintensity compared to the brain, consistent with high ADC values (increased diffusivity) on corresponding ADC map (**e**)

Ultrasound demonstrates a hypoechoic lesion with internal speckled increased echogenicity, often with underlying osseous erosion and internal vascularity on color Doppler [48]. As with other osseous lesions, CT and MRI provide complementary information. The osteolytic LCH lesions are well-defined with ‘’punched-out’’ appearance without periosteal reaction, most commonly and characteristically arising at the superior lateral (temporolateral) orbital rim (affecting the lateral wall and the roof) [1, 47] (Fig. 32). Sclerotic borders and a beveled edge are typical, due to asymmetrical involvement of the inner and outer tables. However, lesions in the early stage may show a more mottled aggressive appearance with irregular periosteal reaction and a wide transition zone, mimicking osteomyelitis and aggressive neoplasms, such as neuroblastoma [1, 47, 48]. MRI features include intermediate to high ADC values with variable, mostly intermediate and possibly heterogenous T1 and T2 signal intensities. T2 hypointense rim is frequently present, and contrast enhancement is usually heterogenous. Central T2 inhomogeneities and high T1 foci can be seen, corresponding to internal necrosis and hemorrhage, respectively [49]. Higher T2 signal and stronger enhancement at the periphery of the lesion and within the surrounding soft tissue, including dural tail, may be distinctive features of orbital LCH [43, 47, 49] (Fig. 33). In contrast to neuroblastoma and myeloid sarcoma the lesions are rarely bilateral [47]. Orbital LCH may be associated with hypothalamic-pituitary lesions (thickening of the pituitary stalk, possibly with disappearance of the normal high T1 signal of the posterior pituitary lobe), as well as nongranulomatous (neurodegenerative) CNS lesions [47].

**Fig. 34:**
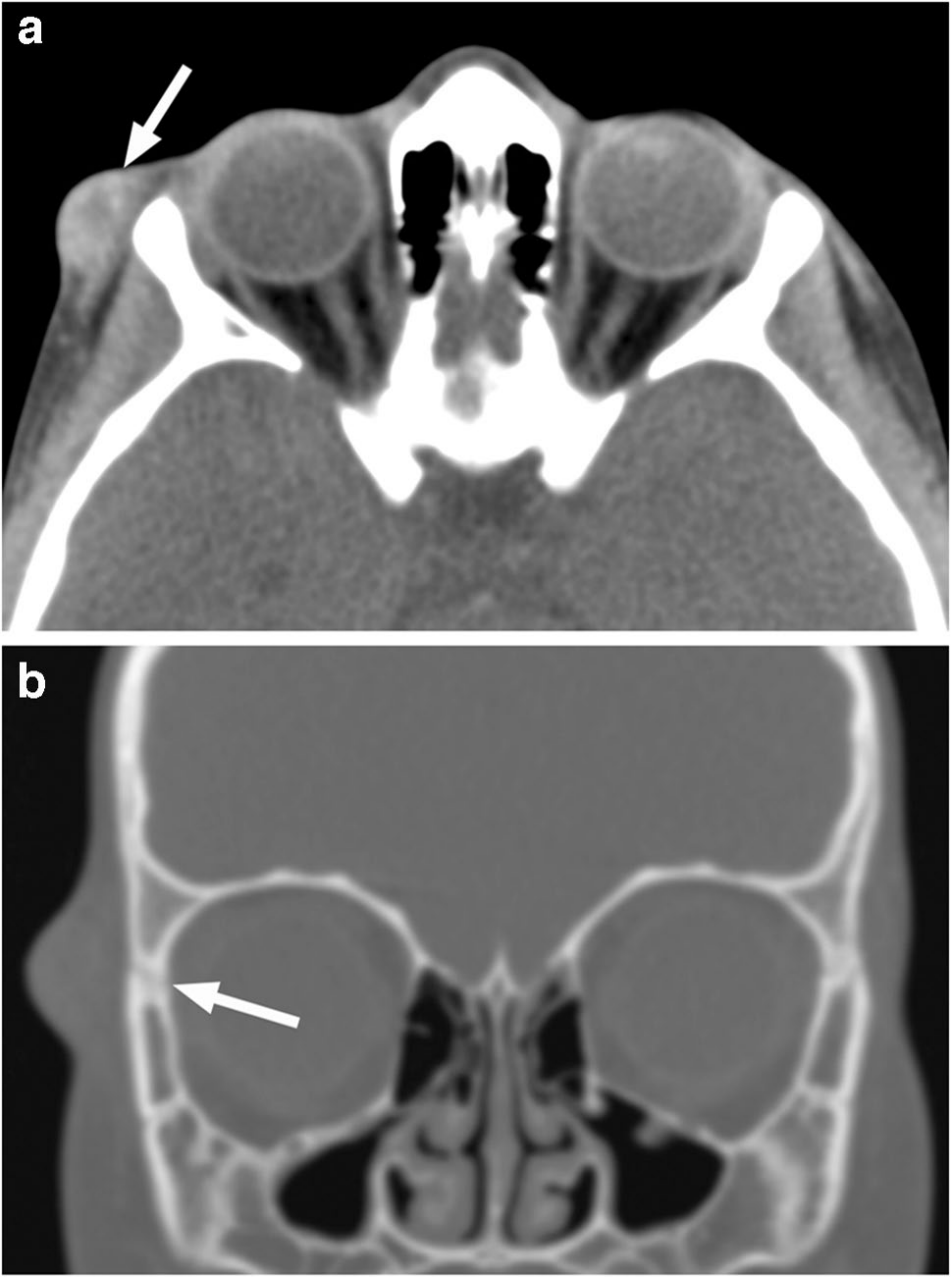
Nonenhanced axial CT image in soft tissue window (**a**) reveals a right periorbital slightly hyperdense oval subcutaneous mass with central hypodensity (arrow). Coronal CT image in bone window (**b**) reveals that the lateral periorbital swelling is adjacent to the right frontozygomatic suture (arrow) in this patient with dermoid

*Dermoids (dermoid cysts)* are the most common orbital cystic tumor of childhood, accounting for up to 46% of all childhood orbital tumors [50]. They are choristomas (histologically normal cells occurring in an abnormal location) that arise from ectoderm trapped in bony sutures during embryogenic migration. They consist of keratinized epithelium and adnexal structures such as hair follicles and sebaceous glands. Epidermoid cysts are similar but lack the adnexal dermal elements [1, 7, 25] and are thought to develop later in embryonic life, after the ectodermal cells become committed to a single cell type [43]. The cysts slowly grow and are usually noticed by the parents during the first year of life as a painless nodule or swelling along the orbital rim but may infrequently arise deeper in the orbit and cause proptosis [1, 51]. Dermoids are characteristically located subcutaneously at the superolateral orbital rim, adjacent to the frontozygomatic suture (Fig. 34), followed by superonasal location [50]. In rare instances they may extend into the orbit (dumbbell-shaped), while other periorbital locations are much less common. Purely intraorbital dermoids are typically found in the superotemporal quadrant (lacrimal fossa) [43, 51]. A well-defined, oval or lobulated lesion in the typical location is consistent with dermoid, and imaging is performed when there is any clinical doubt or concern for intraorbital extension. Treatment is surgical en bloc resection.

**Fig. 35:**
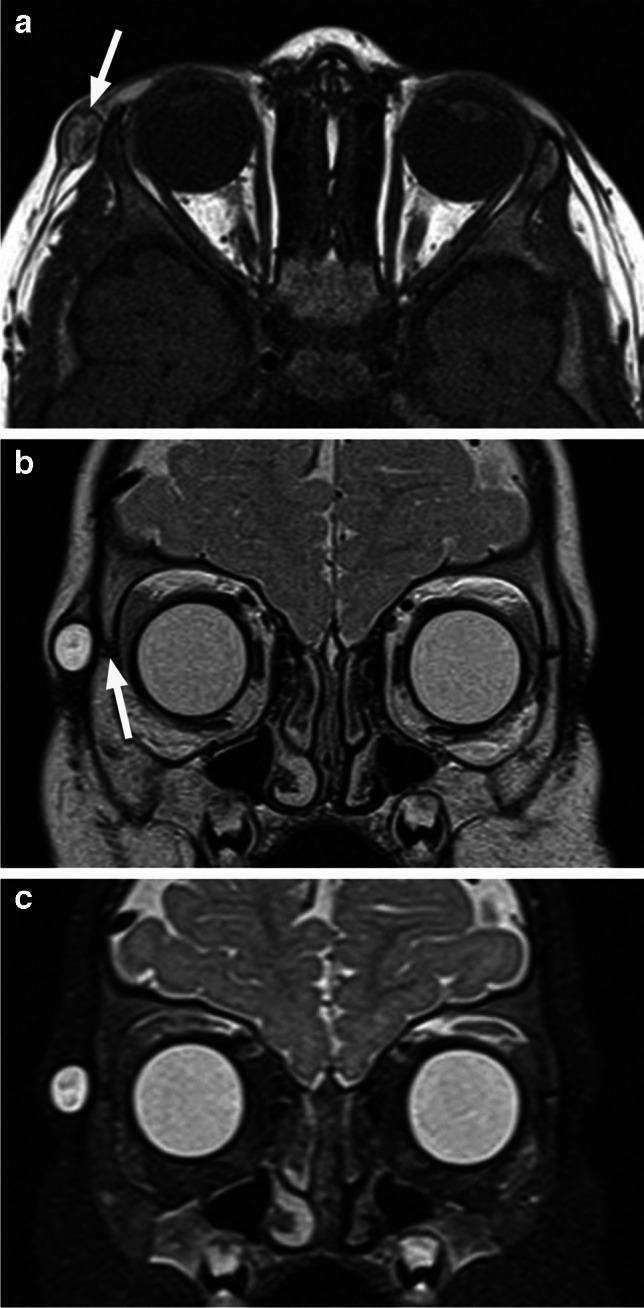
Dermoid. Axial T1-weighted image of the orbits (**a**) shows a well-defined mildly hyperintense oval lesion (arrow) in the typical lateral periorbital location. The mass is hyperintense on coronal T2-weighted image (**b**), without suppression on the corresponding STIR image (**c**). Arrow in (**b**) points to the adjacent frontozygomatic suture

**Fig. 36:**
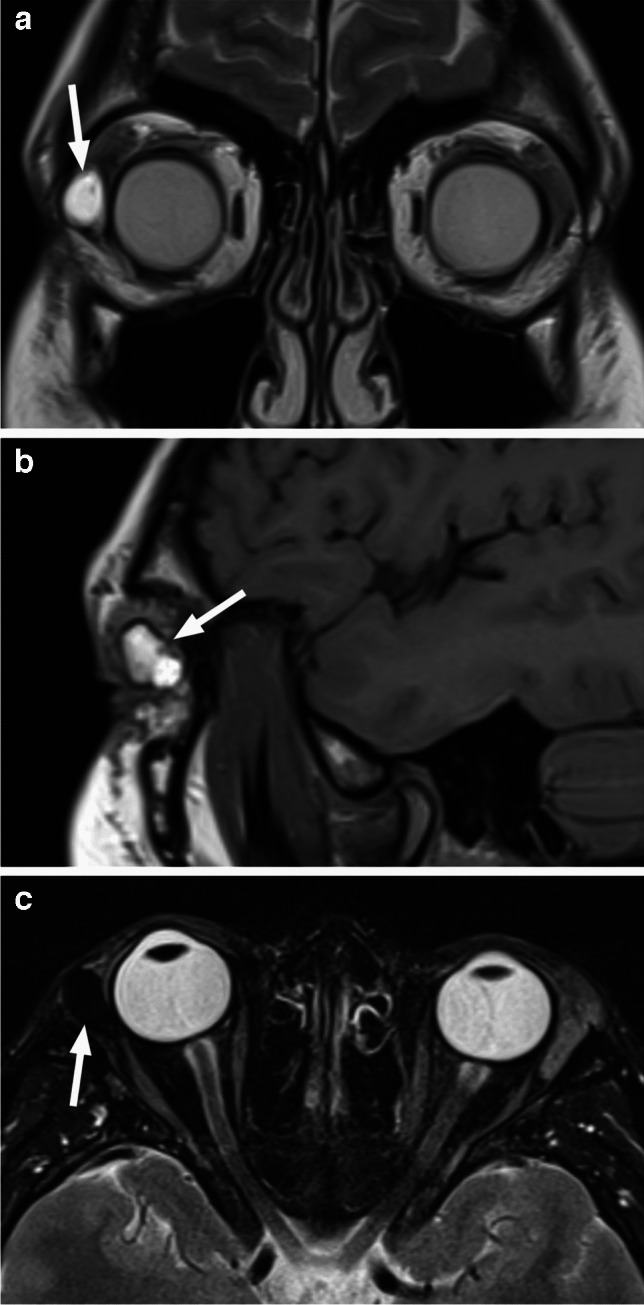
Intraorbital dermoid. Coronal T2-weighted image through the orbits (**a**) shows an oval extraconal circumscribed mass (arrow) on the right side, adjacent to the lacrimal gland. The lesion exhibits fat-like hyperintensity (arrow) on sagittal T1-weighted image (**b**) with complete signal suppression (arrow) on fat saturated axial T2-weighted image (**c**)

In contrast to their intracranial counterparts, fat-like high T1 signal/CT hypodensity is not a consistent feature of orbital dermoids (Figs. 34 and 35), although it is very frequent. When present, the high T1 signal will be suppressed with fat saturation and STIR sequences (Fig. 36). There is no contrast enhancement of intact dermoid, while rim calcifications and thin wall enhancement may occasionally be present. Bony remodeling of the orbital wall is a common finding with larger lesions [1, 7]. The cysts are sometimes bilateral.

Orbital epidermoids are far less common than dermoids but are more frequently intradiploic [52]. Epidermoid cysts are typically comparable to cerebrospinal fluid on CT, T1 and T2-weighted MR sequences (T1 hypointense and T2 hyperintense, without fat on CT), characteristically very bright on DWI. Other pathologies, such as fibrous dysplasia, ossifying fibroma and nodular fasciitis [29, 43, 48] are not included in this review.

## Conclusion

Imaging plays an important role for the diagnosis and treatment planning of pediatric orbital mass lesions and compartment-based approach is very helpful for radiological assessment, as there are only a few most common pathologies in each of the 4 spaces (intraocular, intraconal, extraconal and bony walls), although some diseases may be trans-spatial, primarily vascular malformations. MRI is the imaging modality of choice, which may be complemented with CT for osseous changes. By analyzing and combining the characteristic imaging appearances, including specific location, lesion margins, internal morphology, ADC values and contrast enhancement pattern, the correct diagnosis should be made in most cases.
